# Advancing ^89^Zr-immuno-PET in neuroscience with a bispecific anti-amyloid-beta monoclonal antibody - The choice of chelator is essential

**DOI:** 10.7150/thno.73509

**Published:** 2022-10-09

**Authors:** Thomas E. Wuensche, Natascha Stergiou, Iris Mes, Mariska Verlaan, Maxime Schreurs, Esther J. M. Kooijman, Bart Janssen, Albert D. Windhorst, Allan Jensen, Ayodeji A. Asuni, Benny Bang-Andersen, Wissam Beaino, Guus A. M. S. van Dongen, Danielle J. Vugts

**Affiliations:** 1 Amsterdam UMC location Vrije Universiteit Amsterdam, dept Radiology & Nuclear Medicine, De Boelelaan 1117, Amsterdam, The Netherlands.; 2 Amsterdam Neuroscience, Brain imaging, Amsterdam, The Netherlands.; 3 H. Lundbeck A/S, Ottiliavej 9, 2500 Valby, Denmark

**Keywords:** Aducanumab, Amyloid-beta, Transferrin receptor, ^89^Zr-immuno-PET, DFO*

## Abstract

The accelerated approval of the monoclonal antibody (mAb) aducanumab as a treatment option for Alzheimer's Disease and the continued discussions about its efficacy have shown that a better understanding of immunotherapy for the treatment of neurodegenerative diseases is needed. ^89^Zr-immuno-PET could be a suitable tool to open new avenues for the diagnosis of CNS disorders, monitoring disease progression, and assessment of novel therapeutics. Herein, three different ^89^Zr-labeling strategies and direct radioiodination with ^125^I of a bispecific anti-amyloid-beta aducanumab derivate, consisting of aducanumab with a C-terminal fused anti-transferrin receptor binding single chain Fab fragment derived from 8D3 (Adu-8D3), were compared *ex vivo* and *in vivo* with regard to brain uptake and target engagement in an APP/PS1 Alzheimer's disease mouse model and wild type animals.

**Methods:** Adu-8D3 and a negative control antibody, based on the HIV specific B12 antibody also carrying C-terminal fused 8D3 scFab (B12-8D3), were each conjugated with NCS-DFO, NCS-DFO*, or TFP-*N*-suc-DFO-Fe-ester, followed by radiolabeling with ^89^Zr. ^125^I was used as a substitute for ^124^I for labeling of both antibodies. 30 µg of radiolabeled mAb, corresponding to approximately 6 MBq ^89^Zr or 2.5 MBq ^125^I, were injected per mouse. PET imaging was performed 1, 3 and 7 days post injection (p.i.). All mice were sacrificed on day 7 p.i. and subjected to *ex vivo* biodistribution and brain autoradiography. Immunostaining on brain tissue was performed after autoradiography for further validation.

**Results:** *Ex vivo* biodistribution revealed that the brain uptake of [^89^Zr]Zr-DFO*-NCS-Adu-8D3 (2.19 ±0.12 %ID/g) was as high as for its ^125^I-analog (2.21 ±0.15 %ID/g). [^89^Zr]Zr-DFO-NCS-Adu-8D3 and [^89^Zr]Zr-DFO-*N*-suc-Adu-8D3 showed significantly lower uptake (< 0.65 %ID/g), being in the same range as for the ^89^Zr-labeled controls (B12-8D3). Autoradiography of [^89^Zr]Zr-DFO*-NCS-Adu-8D3 and [^125^I]I-Adu-8D3 showed an amyloid-beta related granular uptake pattern of radioactivity. In contrast, the [^89^Zr]Zr-DFO-conjugates and the control antibody groups did not show any amyloid-beta related uptake pattern, indicating that DFO is inferior for ^89^Zr-immuno-PET imaging of the brain in comparison to DFO* for Adu-8D3. This was confirmed by day 7 PET images showing only amyloid-beta related brain uptake for [^89^Zr]Zr-DFO*-NCS-Adu-8D3. In wild type animals, such an uptake was not observed. Immunostaining showed a co-localization of all administered Adu-8D3 conjugates with amyloid-beta plaques.

**Conclusion:** We successfully demonstrated that ^89^Zr-immuno-PET is suitable for imaging and quantifying amyloid-beta specific brain uptake using a bispecific aducanumab brain shuttling antibody, Adu-8D3, but only when using the novel chelator DFO*, and not DFO, for labeling with ^89^Zr.

## Introduction

Despite the controversy around its efficacy, the recent approval by the U.S. Food and Drug Administration (FDA) of ADUHELM™ (monoclonal antibody (mAb) aducanumab) as a treatment for Alzheimer's Disease (AD) has confirmed the potential of immunotherapy for the treatment of neurodegenerative diseases 1. Parallels already exist in several other indications, with numerous mAb based immunotherapeutics approved by the FDA 2. In addition to the progress made with these novel biological entities, the field of immuno-positron emission tomography (immuno-PET) has come to prominence. By combining the high affinity and specificity of mAbs with the non-invasive imaging technique of PET, this method has become an attractive tool in drug development in recent years, e.g., for patient selection 3. Advancing into the field of central-nervous-system (CNS) diseases, immuno-PET has the potential as a complementary theranostic tool to open new avenues for diagnosis, monitoring disease progression, and therapy response 4.

The main obstacle for using mAbs as a therapeutic, and therefore also for establishing immuno-PET for CNS diseases, is the limited passive transport of these large molecules across the blood-brain barrier (BBB) 5. Previous work by Fissers *et al*. and Waldron *et al.* showed that a ^89^Zr-radiolabeled monoclonal anti-amyloid antibody *[^89^Zr]-Df-Bz-JRF/A*β*N/25* indeed binds to amyloid plaques *in vivo*. However, low brain penetrance and high non-specific binding limited its usefulness as a plaque imaging agent 6,7. It was suggested that strategies to enhance brain uptake would lead to better results. In recent years, this general issue has been tackled with several approaches, either by modifying the BBB permeability (e.g., osmotic opening or focused ultrasound) or by altering the protein itself for enhanced uptake. One of these methods is the attachment of a “molecular trojan horse'', which enables a higher brain uptake due to receptor-mediated transcytosis (RMT) across the BBB 8. Recently, several amyloid-beta mAbs have been modified with the scFv variant of the murine antibody 8D3, monovalently binding to the murine transferrin receptor 1 (mTfR1) for enhanced brain uptake. Using this system, several successful preclinical ^124^I-immuno-PET applications were recently published 9-11. With a half-life of 4.2 days, PET imaging with iodine-124 is possible for up to a week, ensuring decent target-to-background ratios. Still, this radionuclide has limitations concerning routine availability and high-resolution imaging, making it less attractive for clinical applications. In contrast, ^89^Zr has a comparable half-life of 3.3 days whilst being broadly available and clinically applied and having better physical characteristics for PET imaging 12,13.

**Figure 1:**
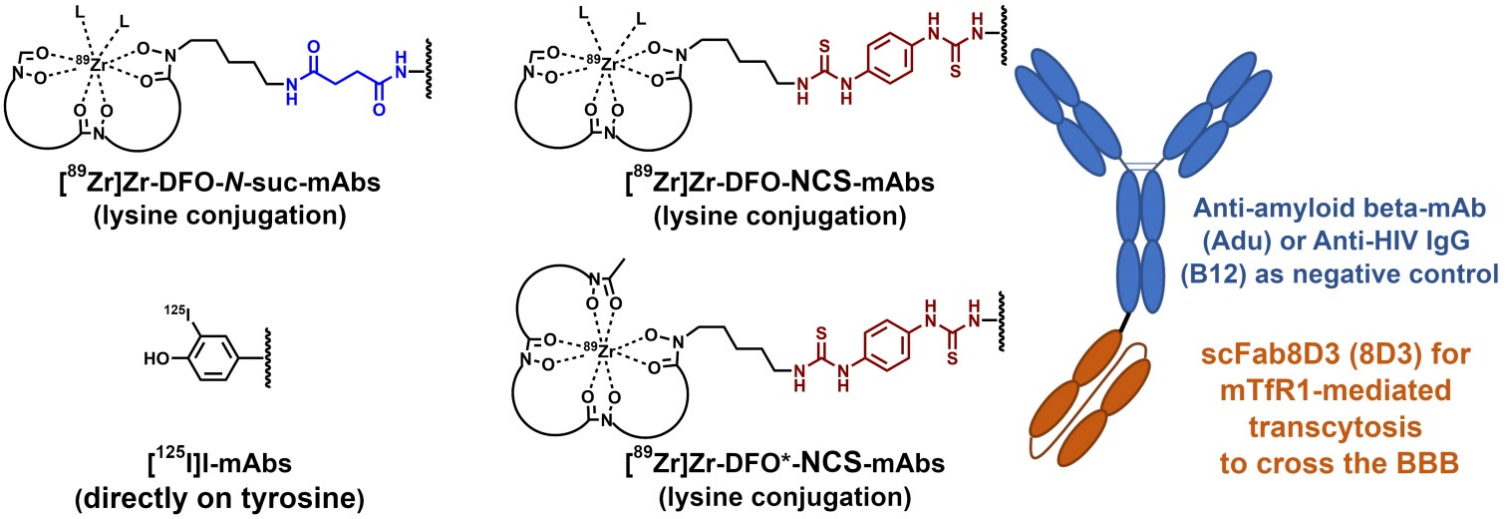
Overview of the four types of radiolabeling methods applied to the two bispecific antibodies.

Taking these considerations into account, ^89^Zr-labeled bispecific monoclonal antibodies, modified for mTfR1-mediated transcytosis via a C-terminal fused anti-transferrin receptor binding single chain Fab fragment derived from 8D3, seem an attractive approach to advance the use of ^89^Zr-immuno-PET into the field of CNS diseases. Therefore, our aim was to establish ^89^Zr radiochemistry for brain application by performing *ex vivo* and *in vivo* biodistribution studies of a bispecific anti-amyloid-beta aducanumab derivate mAbAdu-scFab8D3 (Adu-8D3), in an APP/PS1 mouse model of Alzheimer's Disease. We used the residualizing radionuclide ^89^Zr in combination with three different bifunctional chelators, including the clinically used chelators DFO-NCS and DFO-*N*-suc 14,15 and the recently developed chelator DFO*-NCS 16 for labeling of Adu-8D3 and compared it to non-residualizing ^125^I (as a substitute for ^124^I) (**Figure 1**). We evaluated the brain uptake and target engagement of radiolabeled Adu-8D3 *ex vivo* and* in vivo* by PET imaging of APP/PS1 TG mice, using a bispecific anti-HIV-monoclonal antibody mAbB12-scFab8D3 (B12-8D3) and wild type animals as controls.

## Results

### ^89^Zr- and ^125^I-labeling of Adu-8D3 and control B12-8D3 and *in vitro* binding

To ensure high specific activity, we adapted our standard conjugation procedures by using higher equivalents of DFO-NCS, DFO*-NCS, or TFP-*N*-suc-DFO-Fe-ester during mAb conjugation, with longer reaction times and smaller reaction volumes. In the subsequent radiolabeling with ^89^Zr, a constant specific activity of 0.2 MBq/µg protein for all formulated products was achieved. The ^125^I-labeling was performed via electrophilic aromatic substitution on tyrosine, using a modified iodogen-coated mAb method. This method allowed radioiodinations with minor amounts of iodogen, ensuring mild radiolabeling conditions 17.

**Figure 2:**
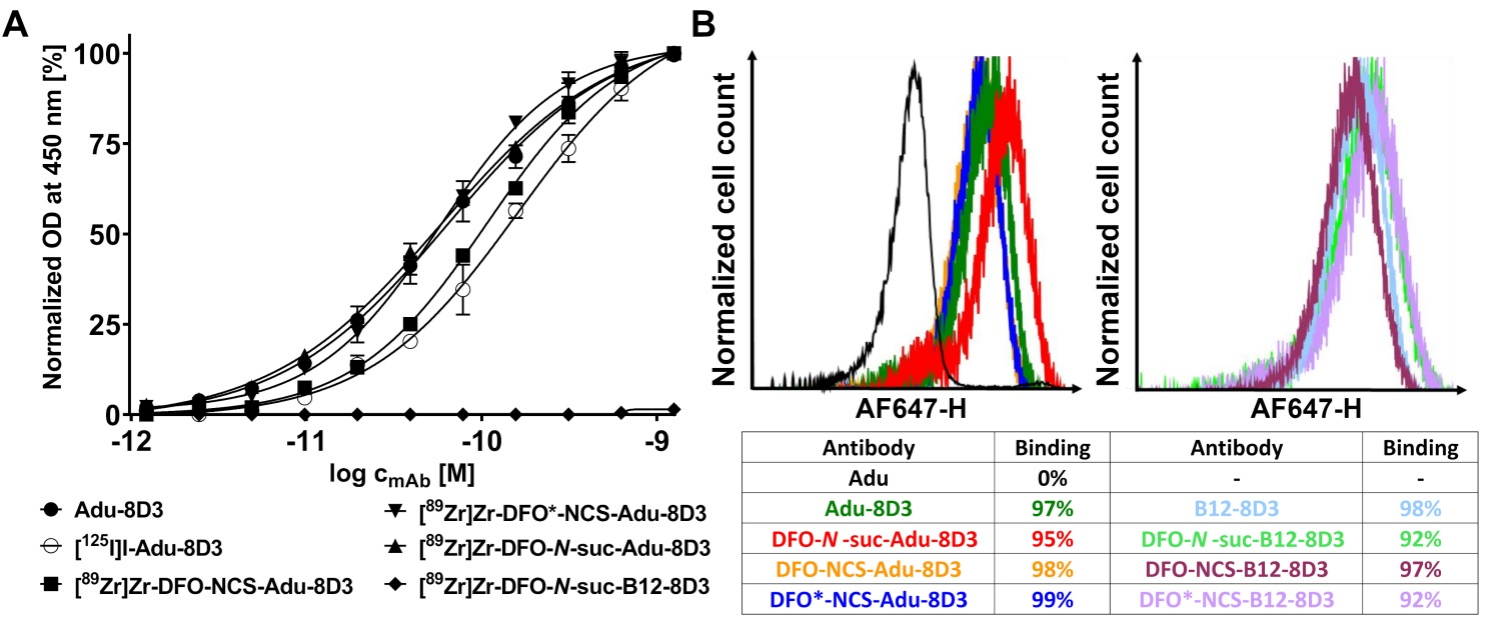
***In vitro* binding assessment via ELISA and FACS. A)** Indirect amyloid-beta Var24 (amyloid-beta vaccine construct consisting of three amyloid-beta 1-12 peptides) ELISA with all four radiolabeled Adu-8D3 constructs, unlabeled Adu-8D3, and [^89^Zr]Zr-B12-8D3 as a negative control. **B)** FACS analysis of the six non-radiolabeled DFO/DFO* modified constructs and three non-modified constructs, with Adu being the negative control, assessed with mTfR1 transfected CHO-S cells.

To evaluate the influence of mAb modification and radiolabeling on the binding to amyloid-beta, ELISA was performed with all radiolabeled Adu-8D3 constructs using [^89^Zr]Zr-DFO-*N*-suc-B12-8D3 as negative control; no impairment of affinity to amyloid-beta was observed for all radiolabeled Adu-8D3 conjugates, and no binding of B12-8D3 to amyloid-beta (**Figure 2A**) was observed. The binding towards the mTfR1 was determined via FACS analysis of the non-radiolabeled DFO/DFO*-modified mAbs, and none of the constructs showed impaired binding to mTfR1 (**Figure 2B**).

### Brain uptake of ^89^Zr- and ^125^I-labeled Adu-8D3 and control B12-8D3 in APP/PS1 TG mice

**Figure 3:**
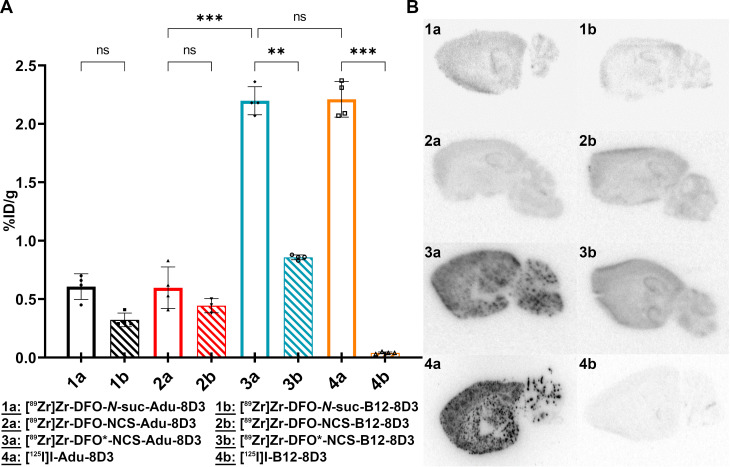
***Ex vivo* brain uptake and brain distribution.** 11-13 month old APP/PS1 TG mice were injected with 30 µg of the radioimmunoconjugates, and *ex vivo* analysis was performed on day 7 p.i.; The brain uptake is given as %ID/g (mean ±SD, n = 3-5 animals per group). **A)** *Ex vivo* brain uptake of all eight groups. Significant differences between the groups are marked with asterisks (**p < 0.01; ***p < 0.001) or marked as non-significant (ns). **B)** Autoradiography of 20 µm cryo-sections, exposure time being 2 weeks for the ^89^Zr-groups and 4 days for the ^125^I-groups.

**Figure 4:**
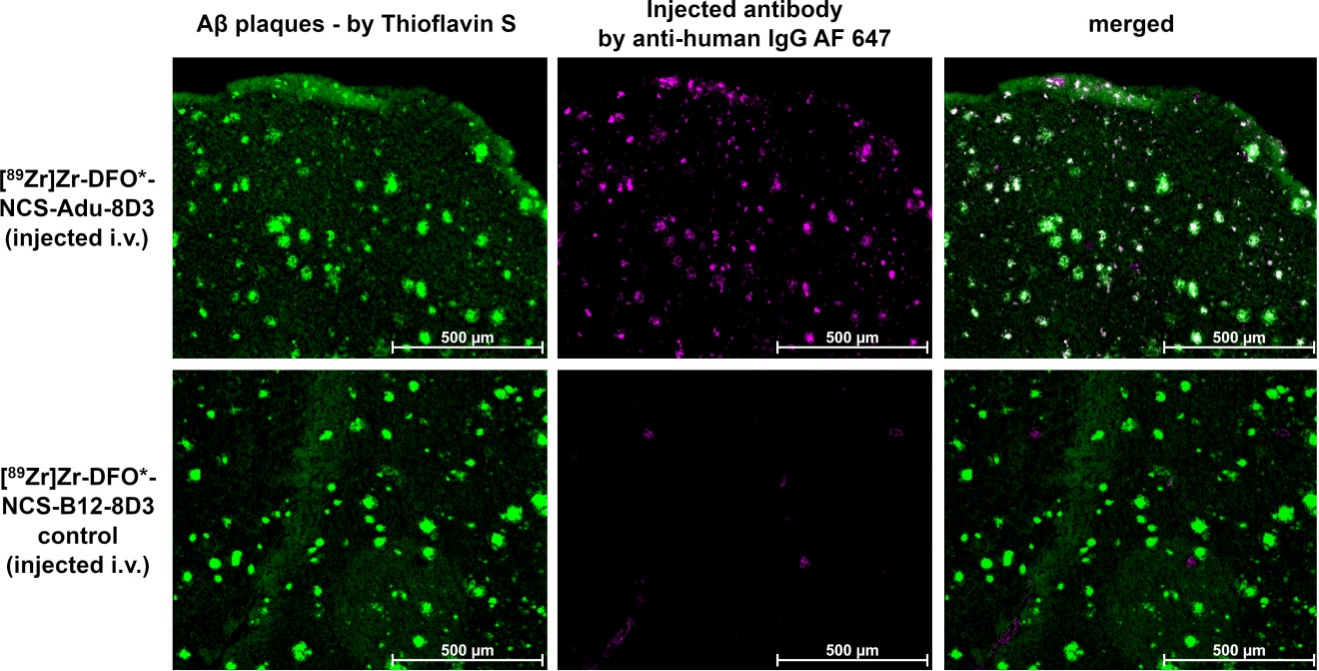
** Immunofluorescence analysis of brain sections of APP/PS1 TG mice.** 11-13 month old APP/PS1 TG mice were injected with a fixed dose of 30 µg of radiolabeled protein. The same 20 µm cryo-sections used for autoradiography (Figure 3) were stained with 0.125% Thioflavin S (yellow) and AF647-goat anti-human IgG (1:1000, purple) to detect injected antibody. Shown are the images of each separate and the merged channels, overlay of the two signals appears in white.

Adu-8D3 and control B12-8D3 were radiolabeled with ^89^Zr (three methods) or ^125^I as described above (**Table 1**), and ~30 µg of radiolabeled mAb were injected via the tail vein into APP/PS1 TG mice. Detailed information on age, gender, and weight of the mice in all groups is given in **Table S1**. At day 7 p.i., all mice were sacrificed, and *ex vivo* biodistribution analysis was performed, using the left cerebral hemisphere to determine the brain uptake (**Figure 3A**), while also uptake in other organs was assessed (**Table S2-S5** and **Figure S1-S4**). The right cerebral hemispheres were frozen, and 20 µm sections were cut to perform autoradiography (**Figure 3B**) and immunofluorescence staining (**Figure 4**). In addition, an analogous experiment was done with the monoclonal aducanumab derivative (without the 8D3 modification), radiolabeled with iodine-125, of which the same detailed information as for the other groups is given in **Table S1**, **Table S5** and **Figure S4**.

The brain uptake of [^89^Zr]Zr-DFO*-NCS-Adu-8D3 (2.19 ±0.12 %ID/g) was comparable to [^125^I]I-Adu-8D3 (2.21 ±0.15 %ID/g) in 11-13 month old APP/PS1 TG mice. In contrast, both the [^89^Zr]Zr-DFO-NCS- and the DFO-*N*-suc-Adu-8D3 analogs showed a significantly lower brain uptake (< 0.65 %ID/g). Furthermore, control conjugate [^89^Zr]Zr-DFO*-NCS-B12-8D3 showed a significantly lower brain uptake (0.86 ±0.15 %ID/g) than [^89^Zr]Zr-DFO*-NCS-Adu-8D3, being comparable to the uptake of [^89^Zr]Zr-DFO-NCS- and [^89^Zr]Zr-DFO-*N*-suc-Adu-8D3 (**Figure 3A**). As expected, the uptake level of control [^125^I]I-B12-8D3 in the brain was very low (< 0.05 %ID/g). To confirm the efficacy of the mTfR1 mediated BBB transcytosis 8D3 modification, [^125^I]I-Adu-8D3 was compared to the unmodified monoclonal antibody [^125^I]I-Adu and showed an 8.8 fold higher brain uptake (**Table S1**). *Ex vivo* autoradiography at day 7 p.i. showed a granular uptake pattern of radioactivity, similar to plaques distribution, only for [^89^Zr]Zr-DFO*-NCS- and [^125^I]I-Adu-8D3, while the two [^89^Zr]Zr-DFO-conjugates and the controls showed no such uptake pattern (**Figure 3B**).

Remarkable differences in radioactivity uptake (Table S2-S5 and Figure S1-S4) between the ^89^Zr and ^125^I groups were observed for several organs, with ^89^Zr showing higher uptake values in, e.g., liver, spleen, kidney and boney tissue and ^125^I in the thyroid.

Immunostaining using Thioflavin S to detect amyloid-beta plaques in combination with a secondary antibody to detect the injected mAb conjugates revealed that the injected Adu-8D3 (independent of the radiolabeling procedure) co-localized with amyloid-beta, whilst for all B12-8D3 control groups, no or negligible injected antibody was detectable. **Figure 4** shows representative images for the [^89^Zr]Zr-DFO*-NCS-Adu-8D3 and its respective control; the complete data set is given in **Figure S5**. These results confirm that specific delivery of the radionuclide to the brain target is only possible if Adu-8D3 is radiolabeled with [^89^Zr]Zr-DFO* or ^125^I.

**Figure 5:**
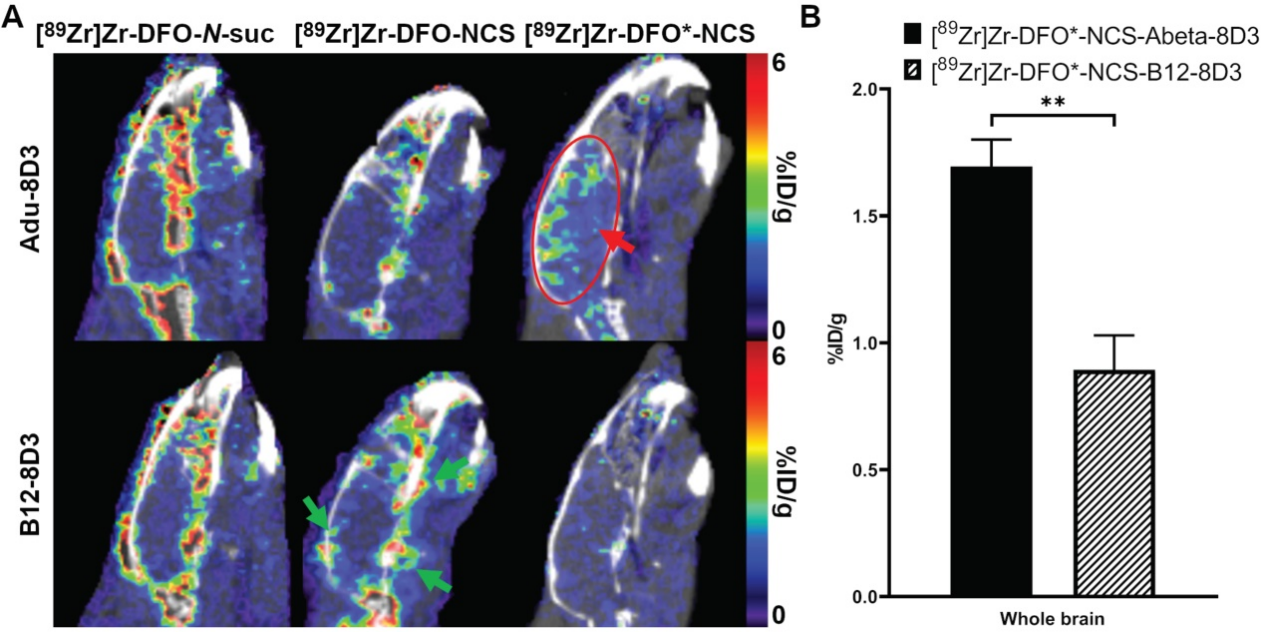
** Brain uptake of [^89^Zr]Zr-Adu-8D3 and control B12-8D3 mAbs using three different chelators in APP/PS1 TG mice as assessed by PET/CT.** 11-13 month old APP/PS1 TG mice were injected with 30 µg of the radioimmunoconjugates and imaged with PET/CT at day 7 p.i. **A)** One sagittal representative PET/CT image is shown per group. The red arrow and circle indicate the amyloid-beta related brain uptake for [^89^Zr]Zr-DFO*-NCS-Adu-8D3, while the green arrows in the picture of the DFO control exemplifies the difference in uptake around the brain for the four DFO groups in contrast to DFO*. **B)** PET brain quantification. Significant differences between the groups are marked with asterisks (**p < 0.01).

Biodistribution of the ^89^Zr-labeled mAbs was further evaluated by PET imaging at day 7 p.i. The sagittal PET images (**Figure 5A**) show high brain uptake with localized hotspots only for [^89^Zr]Zr-DFO*-NCS-Adu-8D3 (red arrow and circle). For the two ^89^Zr-DFO groups and all three control groups, no such signal was recognizable. PET quantification was performed using the brain atlas tool. However, the analysis appeared to be hampered for the DFO groups, most likely due to spill-in from areas around the brain (exemplified by green arrows in **Figure 5A**). In contrast, no significant spill-in for the two DFO* groups was observed (**Figure 5A**), allowing accurate quantification (**Figure 5B**). Uptake at day 7 p.i. was 1.70 ±0.12 %ID/g for [^89^Zr]Zr-DFO*-NCS-Adu-8D3 and 0.89 ±0.14 %ID/g for [^89^Zr]Zr-DFO*-NCS-B12-8D3 for the whole brain, correlating well with the *ex vivo* biodistribution results. Representative whole body PET/CT MIP images for all *in vivo* study groups day 7 p.i. are given in **Figure S11**.

### Brain uptake of [^89^Zr]Zr-DFO*-NCS-Adu-8D3 and [^125^I]I-Adu-8D3 in wild type mice

To further investigate the amyloid-beta targeting specificity of [^89^Zr]Zr-DFO*-NCS-Adu-8D3 and [^125^I]I-Adu-8D3, as seen in APP/PS1 TG mice, the compounds were also evaluated by direct comparison of brain uptake in APP/PS1 TG and age-matched wild type (WT) mice.

**Figure 6:**
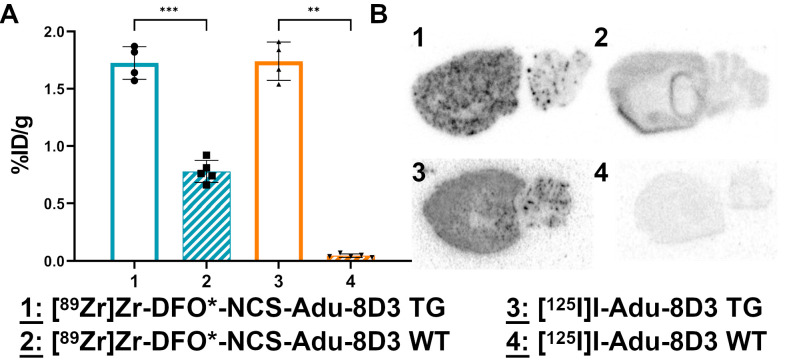
*Ex vivo* brain uptake and brain distribution of [^89^Zr]Zr-DFO*-NCS-Adu-8D3 and [^125^I]I-Adu-8D3 in APP/PS1 TG and wild type mice. 11-13 month old male TG or WT mice were injected with 30 µg of the radioimmunoconjugates, and *ex vivo* analysis was performed on day 7 p.i.; The brain uptake is given as %ID/g (mean ±SD, n = 4-5 animals per group). **A)** *Ex vivo* brain uptake. Significant differences between the groups are marked with asterisks (**p < 0.01;***p < 0.001). **B)** Autoradiography of 20 µm cryo-cuts, 2 weeks exposure time for the ^89^Zr-groups and 4 days for the ^125^I-groups.

The *ex vivo* brain uptake of [^89^Zr]Zr-DFO*-NCS-Adu-8D3 (1.73 ±0.14 %ID/g) was again comparable to [^125^I]I-Adu-8D3 (1.74 ±0.17 %ID/g) in APP/PS1 TG mice (**Figure 6A**). Uptake in brains of wild type animals corresponded well to the B12-8D3 control groups in APP/PS1 TG mice (**Figure 3A**), showing a relatively high uptake for [^89^Zr]Zr-DFO*-NCS-Adu-8D3 and a low uptake for [^125^I]I-Adu-8D3. Differences in uptake of ^89^Zr and ^125^I in other organs appeared to be the same as observed before (**Table S4** and **Figure S3**).

### Brain uptake kinetics of [^89^Zr]Zr-DFO*-NCS-Adu-8D3

**Figure 7:**
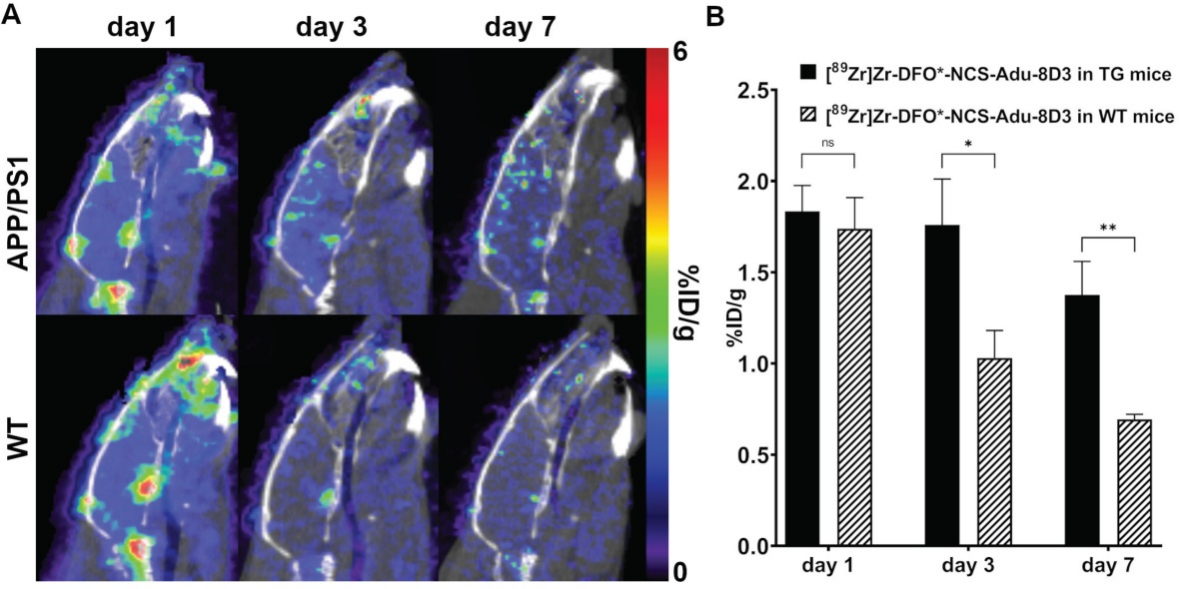
Brain uptake of [^89^Zr]Zr-DFO*-NCS-Adu-8D3 at day 1, 3 and 7 p.i. in APP/PS1 TG and WT mice as assessed by PET/CT. 11 month old male APP/PS1 TG or WT mice were injected with 30 µg of the radioimmunoconjugate. **A)** One representative sagittal PET/CT image is shown per group. **B)** PET brain quantification for the whole brain. Significant differences between the groups are marked with asterisks (*p < 0.05, **p < 0.01; ns, non-significant).

PET imaging with [^89^Zr]Zr-DFO*-NCS-Adu-8D3 in APP/PS1 TG and wild type animals was performed on day 1, 3 and 7 post-injection to evaluate the brain uptake kinetics of the antibody. The images and the quantification revealed that after 24 hours the brain uptake difference is marginal for both groups. On day 3 and 7, significantly different brain uptake was observed between the APP/PS1 TG and WT mice (**Figure 7**).

## Discussion

We investigated the feasibility of ^89^Zr-immuno-PET for neuroimaging with two aims: Firstly, to investigate if, analogous to ^89^Zr-immuno-PET imaging in the periphery 18-20, DFO* can be of added value compared to the clinically used bifunctional chelators *N*-suc-DFO and DFO-NCS. To test this, the bispecific anti-amyloid-beta aducanumab derivate mAbAdu-scFab8D3 (Adu-8D3) was used. This bispecific antibody was designed to overcome the low passive BBB permeability of monospecific mAbs, which have shown limited ^89^Zr-immuno-PET potential for imaging amyloid-beta in the past 6,7. The use of this bispecific mAb, consisting of an amyloid-beta targeting aducanumab derivate and the BBB shuttling moiety 8D3, for immuno-PET imaging, has previously been established by Syvänen *et al*. using iodine-124 as the PET radionuclide 9-11. Despite having an ideal half-life for immuno-PET, iodine-124 suffers from limited availability, high costs, and high positron energy which limits high resolution imaging 21,22. Therefore, the second aim was to compare the residualizing radiometal zirconium-89 to the non-residualizing iodine-125 (as a substitute for iodine-124) by comparison of the biodistribution of the respective radiolabeled Adu-8D3.

To achieve both aims, we used the APP/PS1 Alzheimer's disease mouse model, paired with the bispecific anti-HIV-mAb mAbB12-scFab8D3 (B12-8D3) and wild type animals as controls. Adu-8D3 and control B12-8D3 were either modified with the three different chelators, followed by radiolabeling with ^89^Zr, or labeled via direct radioiodination with ^125^I (**Figure 1**). All eight radiolabeled constructs were generated in sufficient quantity and in excellent radiochemical purity with non-impaired binding to their corresponding antigens, guaranteeing a similar starting point for *in vivo* studies (**Figure 2**, **Table 1**).

*Ex vivo* biodistribution and autoradiography of brain tissue revealed that the use of DFO* led to superior results in comparison to the DFO chelators. [^89^Zr]Zr-DFO*-NCS-Adu-8D3 showed a significantly higher brain uptake and a granular pattern of radioactivity similar to plaques distribution, suggesting specific amyloid-beta imaging of the radioconjugate in APP/PS1 TG mice. This was not observed for [^89^Zr]Zr-DFO-Adu-8D3 constructs (NCS and *N*-suc linker), the three [^89^Zr]Zr-B12-8D3 control constructs (**Figure 3**) and [^89^Zr]Zr-DFO*-NCS-Adu-8D3 in wild type animals (**Figure 6A**). These results were confirmed by PET images on day 7 p.i. showing only amyloid-beta related brain uptake with focal hotspots for [^89^Zr]Zr-DFO*-NCS-Adu-8D3 in APP/PS1 TG mice, aligning with the *ex vivo* results (**Figure 5A**).

TfR1-mediated brain uptake of the mAbs under investigation was demonstrated as the uptake was 7 to 8.8 fold higher for [^125^I]I-Adu-8D3 than for monoclonal antibody [^125^I]I-Adu, lacking the BBB shuttling moiety (**Table S5**). The observed increase in brain uptake was in line with previous reports by Syvänen *et al*. 9-11.

[^89^Zr]Zr-DFO*-NCS-Adu-8D3 and [^125^I]I-Adu-8D3 showed similar uptake values and uptake patterns in the brain of APP/PS1 TG mice as measured by *ex vivo* biodistribution (**Figure 3A** and **6A**) and autoradiography (**Figure 3B** and **Figure 6B**). Granularity of the autoradiography seemed more distinct in the case of ^125^I than ^89^Zr, but this is due to the low gamma energy of ^125^I, which is optimally suited for phosphorimager analysis. The significantly lower brain uptake of control [^125^I]I-B12-8D3 in APP/PS1 TG mice and [^125^I]I-Adu-8D3 in WT mice in comparison with the [^89^Zr]Zr-DFO* controls might reflect the non-residualizing character of radioiodine in comparison to the residualizing radiometal (**Figure 3A**). The deiodination processes of radioiodine tracers, when the direct labeling via electrophilic substitution on tyrosine is performed, are very well understood and described and may explain the low brain uptake of our ^125^I control groups 23. For ^89^Zr on the other hand, several comparative studies using ^89^Zr along ^124^I have shown the residualizing character of ^89^Zr 12,24-26, putting it in the same category as other radiometals such as ^177^Lu, ^90^Y and ^111^In. The low brain uptake of our control groups, [^125^I]I-Adu-8D3 in WT mice and [^125^I]I-B12-8D3 in APP/PS1 TG mice, corresponds with the brain uptake of similar compounds in WT mice as described by Syvänen et al. 9-11. A direct comparison of ^125^I-labeled and ^111^In-labeled TfR targeting antibodies was performed by Bien-Ly et al. in wild type mice 27. They observed a similar residualizing characteristic for ^111^In and a non-residualizing characteristic for ^125^I as we did for ^89^Zr and ^125^I. Possible reasons for the observed higher brain uptake could be glial cell related uptake and subsequent phagocytosis or the expression of TfR on neurons 28,29, leading to neuronal internalization and retention of ^89^Zr. In the latter case, it was recently suggested by Roshanbin et al. that residualizing radiometals, such as ^89^Zr, could even be beneficial to target intracellular alpha-synuclein pathology 30.

The difference in residualizing behavior of the two used radionuclides was also observed in other organs. ^89^Zr showed significantly higher uptake levels in, e.g., liver, spleen, kidney and boney tissue. Due to the TfR specificity of used constructs, the higher uptake of ^89^Zr in the spleen is most probably related to the high TfR expression in this organ 31. Higher uptake in the kidney and liver can be explained by the fact that those are metabolic organs, resulting in a longer retention time of ^89^Zr after catabolic degradation 32. Similar to the spleen, uptake in boney tissue could be explained by the erythrocyte progenitor cells in the bone marrow, which express high levels of TfR, leading to cellular uptake and subsequent degradation. In addition, the well known tropism of free ^89^Zr for bones, due to the affinity to apatite, can contribute to the accumulation of free ^89^Zr in the bone independent of the constructs used. In the case of radioiodine, the expected high accumulation in the thyroid after elimination is observed 23, which doesn't occur with ^89^Zr.

The reason for the observed difference in ^89^Zr brain uptake between DFO* and DFO is not fully understood. One hypothesis is based on stability issues previously observed when comparing ^89^Zr-DFO*-mAbs with ^89^Zr-DFO-mAbs in tumor-bearing mice. While tumor uptake was similar with both constructs, a remarkable higher bone uptake of ^89^Zr was observed in case of ^89^Zr-DFO-mAb, indicative for instability of ^89^Zr-DFO *in vivo*. Although the deposition of ^89^Zr in bone has not been routinely observed in the published clinical ^89^Zr-immuno-PET studies to date, many efforts have been invested in developing chelators with increased *in vivo* stability 33. From an inorganic chemistry perspective, DFO is not the ideal coordinating molecule for zirconium because DFO is a hexadentate chelator consisting of three hydroxamate moieties, while Zr^4+^ prefers forming octadentate complexes. With this in mind, the octadentate analog of DFO consisting of four hydroxamate moieties, called DFO* (“DFO star”), was developed 16. In line with these previous observations, it can be envisioned that the ^89^Zr-DFO* complex is more stable in the physiological environment of the brain than the ^89^Zr-DFO complex. The physiological brain environment is different from the blood and plasma, and therefore the stability of the radioimmunoconjugates could be different in the brain. The interstitial fluid and cerebrospinal fluid (CSF) have similar compositions but differ significantly from blood plasma, mostly in protein concentration 34. Preliminary *in vitro* analyses at our lab, including the incubation of [^89^Zr]Zr-DFO*-mAbs and [^89^Zr]Zr-DFO-mAbs in artificial CSF and porcine CSF, have not yet given indications that the stability of the radioimmunoconjugates could explain the observed differences between DFO and DFO*.

Furthermore, the elevated occurrence of free iron species in the APP/PS1 TG mice 35, resulting in a competition of Fe^3+^ with Zr^4+^ for DFO*/DFO, could be an additional factor. Although no conclusive evidence has yet been found that Fe^3+^ has an influence on the stability of [^89^Zr]Zr-DFO-radioimmunoconjugates *in vivo* to this date, previous *in vitro* experiments have shown such an influence 36,37. This aligns with the high affinity of DFO for Fe^3+^ and its related use in iron chelation therapy 38 and radiolabeling with ^59^Fe 12. Prior work from Chomet *et al.* supports this as it did show that [^89^Zr]Zr-DFO had a slightly lower stability compared to [^89^Zr]Zr-DFO*, when these complexes were incubated with a 10-fold excess of Fe^3+^^18^.

Another hypothesis for the different brain uptake of DFO vs. DFO* is based on the influence that the transport mechanism across the BBB may have. The occurrence of free iron ions and transferrin in the endosome, in combination with the relatively low pH 31, could result in the instability of [^89^Zr]Zr-DFO-mAbs during BBB transcytosis. However, since for all Adu-8D3 constructs, independent of the bioconjugation, brain uptake and co-localization with amyloid-beta plaques were substantiated by *ex vivo* immunofluorescence staining (**Figure S5**), we reason that it is unlikely that the type of bioconjugation (different mass, charge and lipophilicity) affects the level of mAb uptake in the brain. More studies are needed to fully understand the cause of the observed differences in *in vivo* brain-specific uptake of [^89^Zr]Zr-DFO*- and [^89^Zr]Zr-DFO-mAbs.

This work sets the foundation for further investigations, extending ^89^Zr-immuno-PET to other neurodegenerative diseases in preclinical models as well as clinical translation. In addition, it is crucial to investigate the underlying mechanism between the observed differences in ^89^Zr brain uptake between DFO and DFO*. Such studies will learn whether the DFO* chelator is also preferred when alternatives to the receptor-mediated transcytosis, e.g., focused ultrasound or osmotic opening of the BBB, are used to enhance brain uptake.

## Conclusion

We successfully demonstrated that imaging of specific brain uptake of a bispecific aducanumab brain shuttle antibody Adu-8D3 in APP/PS1 TG mice with ^89^Zr is possible when using the chelator DFO*. Inferior results were observed when DFO (irrespective of the linker) was used as the chelator, presumably because of the instability of the Zr-DFO complex. Comparison of ^89^Zr to radioiodine, which was previously used for immuno-PET brain imaging, showed similar specific uptake values. In contrast, brain uptake and uptake in some normal organs were higher for some ^89^Zr-labeled mAbs than for the ^125^I-labeled derivates, most probably because of the residualizing character of the radiometal. However, taking the availability, costs, and superior image quality of zirconium-89 into account, ^89^Zr-immuno-PET could be a viable option for future neurological applications.

## Methods

### General materials

DMSO, Na_2_CO_3_, oxalic acid were obtained from Sigma-Aldrich, Sucrose and Tween20®-pharmaceutical grade from Merck-Millipore, 1 M HEPES from Invitrogen and phosphate buffered saline from Brunschwig Chemie. ^89^Zr in 1 mol/L oxalic acid and ^125^I in 0.1 mol/L NaOH were obtained from Perkin-Elmer (Boston, USA). Water was distilled and deionized using a MilliQ water filtration system (Millipore, USA). p-Isothiocyanatobenzyl desferrioxamine (p-SCN-Bn-deferoxamine (B-705), abbreviated in this publication as DFO-NCS) was purchased from Macrocyclics Inc. (Dallas, TX, USA). DFO*-NCS was synthesised by Mercachem B.V. (Nijmegen, The Netherlands). TFP-*N*-suc-DFO-Fe-ester was synthesized in-house 14. The ELISA antigen, Var24 (amyloid-beta vaccine construct consisting of three amyloid-beta 1-12 peptides), was provided by H. Lundbeck A/S in Valby, Denmark. All other starting reagents and solvents were obtained from Sigma-Aldrich.

### Antibody constructs

All antibodies were produced by H. Lundbeck A/S in Valby, Denmark. The monospecific antibody aducanumab targets insoluble amyloid-beta plaques and soluble aggregates, e.g., protofibrils/oligomers, but not amyloid-beta monomers. The bispecific antibody Adu-8D3 was also designed with the scFab8D3 moiety, fused on the heavy chain C terminus, to target the murine TfR1 and allow shuttling across the BBB. To assess non-specific uptake, B12-8D3 was produced and used as a control antibody, targeting gp120 of HIV1.

Detailed information on antibody design, production and purification is given in the supplementary information.

### Synthesis of ^89^Zr-labeled compounds

#### DFO-*N*-suc-*Adu*-8D3 and DFO-*N*-suc-B12-8D3

DFO-*N*-suc-Adu-8D3 and DFO-*N*-suc-B12-8D3 were prepared as described previously 14 with modifications in used equivalents of TFP-*N*-suc-DFO-Fe-ester chelator, formulation buffer and conjugation time. 1 mg of either Adu-8D3 (14.5 mg/mL) or B12-8D3 (14.3 mg/mL) were diluted to 4 mg/mL with 0.9% NaCl, which resulted in an total volume of 250 µL. The pH was adjusted to 8.9-9.1 with 0.1 M Na_2_CO_3_ and 4.7 µL TFP-*N*-suc-DFO-Fe-ester in acetonitrile (8 mM, 7.5 eq.) was added. Immediately after adding, the mixture was homogenized via gentle shaking. Afterwards, the reaction mixture was incubated for 1.5 hours at room temperature without shaking. At the end of the incubation, the reaction mixture was adjusted to pH 4.5 with 0.01 M H_2_SO_4,_ and 30 µL of 25 mg/mL EDTA solution was added and incubated for 30 minutes at 35 °C and 550 rpm. The reaction mixture was applied on a PD-10 column (GE Healthcare Life Sciences), and fractions of 0.5 mL were collected by eluting with 50 mM sodium acetate/200 mM sucrose, pH 5.4-5.6 + 0.01% Tween-20 (hereafter called formulation buffer). Each fraction was measured on a Nanodrop Spectrophotometer (ThermoFisher^TM^); fractions with the highest UV-absorbance at 280 nm were pooled, and concentration was determined via size-exclusion high-performance liquid chromatography (SE-HPLC). Shortly before the radiolabeling, a concentration and buffer exchange (0.5 M HEPES) step was performed via spin filtration. Information regarding the chelator-to-mAb ratio can be found in the supplementary information.

#### DFO*/DFO-NCS-Adu-8D3 and DFO*/DFO-NCS-B12-8D3

DFO/DFO*-NCS-Adu-8D3 and DFO/DFO*-NCS-B12-8D3 were prepared as described previously 15,18 with modifications in used equivalents of DFO/DFO* chelator, formulation buffer and conjugation time. 1 mg of either Adu-8D3 (14.5 mg/mL) or B12-8D3 (14.3 mg/mL) were diluted to 4 mg/mL with 0.9% NaCl, which resulted in a total volume of 250 µL. The pH was adjusted to 8.9-9.1 with 0.1 M Na_2_CO_3_ and added to 10 µL (5 mM, 10 eq.) DFO/DFO*-NCS solution in DMSO. Immediately after adding the antibody solution, the solution was pipetted up and down 5 times to ensure rapid homogenization of the reaction mixture. Afterwards, the reaction mixture was incubated in a thermomixer at 37 °C and 550 rpm for 2 hours. At the end of the incubation, the reaction mixture was applied on a PD-10 column (GE Healthcare Life Sciences), and fractions of 0.5 mL were collected by eluting with 50 mM sodium acetate/200 mM sucrose + 0.01% Tween-20, pH = 5.4-5.6 (formulation buffer). Each fraction was measured at a Nanodrop Spectrophotometer (ThermoFisherTM); fractions with the highest UV absorbance at 280 nm were pooled, and the concentration was determined via SE-HPLC. Shortly before the radiolabeling, a concentration and buffer exchange (0.5 M HEPES) step was performed via spin filtration. Information regarding the chelator-to-mAb ratio can be found in the supplementary information.

#### ^89^Zr-labeling

All conjugated mAbs were radiolabeled as follows: 150-250 MBq [^89^Zr]Zr-oxalate in 1 M oxalic acid solution were pipetted into a 1.5 mL Eppendorf vial, and 1 M oxalic acid was added to reach a volume of 150 µL. Next, 67.5 µL 2 M Na_2_CO_3_ were added and reacted for 3 min. Subsequently, 375 µL 1 M HEPES buffer was added. Afterwards, 500 µg of the modified mAb in 0.5 M Hepes were added to the reaction mixture and incubated in a thermomixer at room temperature (RT) and 550 rpm for 1 hour. The reaction mixture was then applied to a PD-10 column, and fractions of 0.5 mL were collected by eluting with formulation buffer. Each fraction was measured with a dose calibrator (Veenstra Instruments); fractions with the highest activity were pooled, and the concentration was determined via SE-HPLC. The yield of the radiolabelling was calculated by the following formula:

Radiochemical yield = Activity_radiolabeled antibody_ x radiochemical purity/Activity_total_ x 100%

Unlabeled mAb and formulation buffer were added to formulate the product to 30 µg mAb with a specific activity of 0.19 to 0.21 MBq/µg mAb in ~150 µL formulation buffer per mouse.

### Synthesis of ^125^I-labeled compounds

#### [^125^I]I-Adu-8D3, [^125^I]I-Adu and [^125^I]I-B12-8D3

[^125^I]I-Adu-8D3, -Adu and -B12-8D3 were prepared using a modified iodogen-coated mAb method 17. Approx. 45 MBq of iodine-125 stock solution were transferred to a 20 mL glass vial, gently shaken continuously during the reaction, and diluted with 0.5 M phosphate buffer. To reach a total reaction volume of 500 µL, the amount of phosphate buffer was calculated as follows:

V_phosphate buffer_ = 500 µL - V_Iodine-125_ - V_Protein_ - V_Iodogen_

Subsequently, 500 µg antibody stock solution was added. The reaction was started by adding 35 µL of freshly prepared iodogen in acetonitrile solution (5 µg/70 µL). After 45 seconds, 35 µL iodogen in acetonitrile (5 µg/70 µL) was added again and reacted for 45 seconds. The reaction mixture was quenched by adding 200 µL ascorbic acid solution (25 mg/mL, pH = 5.0). The labeling efficiency was determined with instant thin-layer chromatography (iTLC). Afterwards, the reaction mixture was applied on a PD-10 column, and fractions of 0.5 mL were collected by eluting with formulation buffer. Each fraction was measured with a dose calibrator (Veenstra Instruments); fractions with the highest activity were pooled, and the concentration was determined via SE-HPLC. Unlabeled mAb was further added to formulate the product to 30 µg (22.5 µg for the monovalent Antibody) mAb with a specific activity of 0.08 to 0.09 MBq/µg mAb in ~150 µL per mouse. No ascorbic acid was added to the formulation since the product was injected 1-4 hours after formulation, and radiolysis was not an issue.

### Quality controls

#### Radiochemical purity and protein concentration and integrity

Protein concentration and integrity were determined by SE-HPLC; detailed information and chromatograms are given in the supplementary information.

Radioimmunoconjugates were checked for their radiochemical purity by spin filter analysis or iTLC. [^89^Zr]Zr-DFO*/DFO-NCS-Adu-8D3 and [^89^Zr]Zr-DFO*/DFO B12-8D3 were analyzed by spin filter analysis following a described procedure 19. A wash buffer was prepared consisting of the formulation buffer containing 5% DMSO. 1 µL of product diluted to 100 µL with wash buffer was pipetted onto a 30 kDa cut-off spin filter (Ultracel YM-30, regenerated cellulose, 30 kDa cut-off, Merck Millipore), which was subsequently centrifuged at 14000 rpm for 7 min (Eppendorf 5430). The filter was then washed with 100 µL of the wash buffer and spun again for 7 min at 14000 rpm before being washed a second time with 100 µL buffer and spun down again at the same settings. Subsequently, the filter and combined filtrate were counted separately in a gamma counter (LKB Wallac Gamma Counter, model 1282 Compugamma CS). Radiochemical purity was determined by calculating the ratio of counts on the filter (with background subtracted) to the total number of counts of the filtrate plus the counts on the filter (with background subtracted).

iTLC analysis of [^125^I]I-Adu-8D3, [^125^I]I-Adu and [^125^I]I-B12-8D3 was performed on silica gel-impregnated glass fiber sheets (Biodex, Cat.# 150-771), with citrate buffer (20 mM, pH = 5.0) as the mobile phase. iTLC analysis of [^89^Zr]Zr-*N*-suc-DFO-Adu-8D3 and [^89^Zr]Zr-*N*-suc-DFO-B12-8D3 was performed using the same stationary phase with citrate buffer (20 mM + 55 mM EDTA, pH = 5.0) + 10% acetonitrile as the mobile phase. For all iTLC analyses, 500 µL of the eluent were placed in an 8.5 mL plastic test tube. After development, the strip was immediately taken out and cut at the intended line to separate the top from the bottom. Both pieces were counted separately in a gamma counter (LKB Wallac Gamma Counter, model 1282 Compugamma CS), and radiochemical purity was determined by calculating the ratio of counts on the bottom part (with background subtracted) to the total number of counts (both parts, with background subtracted)).

#### Antigen binding - amyloid-beta Var24-peptide ELISA

To evaluate the influence of mAb modification and radiolabeling on the binding to amyloid-beta, ELISA was performed on all radiolabeled Adu-8D3 constructs and [^89^Zr]Zr-DFO-*N*-suc-B12-8D3 as the negative control. Costar® Assay 96 well polystyrene plates (flat bottom, half area, high binding) were coated with 50 µL/well of 100 ng/mL amyloid-beta Var24-peptide in 0.1 M borate buffer (pH = 11) at 4 °C overnight. The coating solution was disposed, and the wells were blocked using 150 µL/well of 2% BSA in PBS while shaking the plate at RT and 600 rpm for 2 hours. After disposing the blocking solution, incubation was performed in duplicates with a serial dilution of antibody (0 - 250 ng/mL) in incubation buffer (0.1% BSA in PBS + 0.05% Tween-20) at RT and 600 rpm for 1 hour (11 points horizontally, 1:2 dilutions, 50 µL/well). Subsequently, the supernatant was removed, and the wells were washed three times with 0.1% Tween-20 in PBS and incubated with 50 µL/well of goat anti-human IgG (H+L) cross-absorbed-HRP secondary antibody (Invitrogen, 31412; 0.8 µg/mL in incubation buffer), at RT and 600 rpm for 1 hour. After incubation, the solution was removed, and the wells were washed four times with 0.1% Tween-20 in PBS and one time with deionized water (dH2O) before adding 50 µL/well of 3,3',5,5'-Tetramethylbenzidine (TMB) substrate for 5 to 10 minutes without shaking and under dark conditions. The enzymatic reaction was stopped with 50 µL/well of 0.5 M HCl solution, and the absorbance at 450 nm was measured immediately with a microplate reader (TriStar^2^ multimode reader LB 942, Berthold Technologies). The absorbance of 0 ng/mL primary antibody was used as background value and subtracted from the other values. Values are given in relative absorbance by dividing all values by the highest absorbance value of each row. In the case of the negative control, the highest average absorbance of the unmodified Adu-8D3 was used.

#### Antigen binding - mTfR1 transfected CHO-S cells FACS analysis

Binding of all six B12-8D3-DFO^(^*^)^ and Adu-8D3-DFO^(^*^)^ modified mAbs to murine TfR1 was assessed by FACS. Stably transfected CHO-S cell lines with mTfR1 and mock-transfected CHO-S cells were cultured in ExpiCHO Expression Medium (Gibco, cat# A29100-01) with 1% anti-clumping agent (Gibco, cat# 0010057AE) at a cell concentration between 1x10^5 and 2x10^6 viable cells/mL in shaker flasks at 37 °C, 5% CO_2_. The mTfR1 high-expression cells are grown under selection pressure (12 mg/mL Puromycin dihydrochloride (Merck, cat# P9620-10ML)). To prepare the mTfR1 transfected and mock-transfected CHO-S cells for FACS, cells were harvested, washed 3 times with cold PBS (spin 300xg for 5 min), counted (viable cell count, Cedex Hires) and adjusted to 5x10^6 cells/mL in cold PBS. 100 µL of cells were transferred into a 96-well-multi dish (U bottom) plate (0.5x10^6 cells/well), spun down for 5 min at 400xg at 4 °C, and the supernatant was discarded. The cells were stained with live/dead cell stain (L34963, 405nm, Invitrogen). Therefore, a mastermix of 0.1 µL dye in 100 µL PBS w/o Mg^2+^ and Ca^2+^ per sample was prepared. The cells were incubated on ice for 15 min in the dark and washed 3 times by adding a total of 180 mL cold FACS buffer (250 mL PBS w/o Ca^2+^ and Mg^2+^ + 2 mM EDTA (1 mL 0.5 M), 2% normal goat serum) per well and centrifuged for 5 min with 350xg at 4 °C. The cells were blocked by adding 50 µL 10% normal goat serum containing buffer/well (1xPBS w/o Ca^2+^ and Mg^2+^ + 2 mM EDTA (60 µL 0.5M, 10% normal goat serum), mixed well and incubated on ice in the dark for 15 min. After that, the plate was spun down, and the supernatant was discarded. The non-modified and modified mAbs (Adu, Adu-8D3, B12-8D3). were added in a total volume of 85 µL with a concentration of 1 nM/2x10^5 cells, incubated for 20 min on ice in the dark, 3 times washed as described above with FACS buffer. Secondary antibody (goat anti-human IgG, Jackson, cat. # 109-605-008) diluted 1:400 in FACS buffer was added to wells, incubated for 20 min on ice in the dark and washed 3 times with FACS buffer. The cells were fixed with 100 µL of 4% PFA (BD Fixation buffer, cat# 554655) and incubated for 15 min on ice in the dark. Cells were washed 3 times with FACS buffer and resuspended in 180 µL FACS analysis buffer (1xPBS + 2 mM EDTA, 1% BSA (0.1 g/10 mL) (IgG/protease-free, Jackson # 001-000-162, lot# 138456)). Samples were kept on ice in the dark until FACS analysis using a NovoCyte Quanteon. Cells were gated for live, single cells and the mean fluorescence intensity (MFI AF647). The percentage of binding was determined for each mAb and modified mAb in comparison to the unspecific binding of the secondary antibody conjugated with AF647 (FlowJo 10 software). Plots for the FACS gating strategy are given in **Figure S10**.

### *In vivo* experiments

Animal experiments were performed according to the NIH Principles of Laboratory Animal Care, the European Community Council Directive (2010/63/EU) for laboratory animal care and the Dutch Law on animal experimentation (“Wet op de dierproeven,” Stb 1985, 336). The experimental protocol was validated and approved by the central Dutch national committee for animal experimentation (CCD) and the local committee on animal experimentation of the Amsterdam UMC, Vrije Universiteit Amsterdam. The transgenic C57BL/6J-Tg(Thy1-APPSw-Thy1-PSEN1*L166P)21/Jckr, designated in this paper as APP/PS1 TG mice, carry a transgene insertion for the human Abeta42 39. The female or male APP/PS1 TG mice and the wild type control mice (received at 11 to 13 months old from Charles River, USA) were left for at least one week of acclimation before starting experiments. For all the reported studies, the animals were housed under standard conditions (20-24 °C, 50-70% relative humidity, 12 h light/dark cycles) with a maximum of 4 animals per cage (Makrolon). In addition, they were provided with nesting material, sawdust, tap water and food (Teklad Global 16% Protein Rodent Diet, Harlan, Madison, WI, USA) ad libitum.

### Biodistribution

The biodistribution of the radiolabeled mAbs (Adu, Adu-8D3, B12-8D3) constructs was determined as follows: 30 µg radiolabeled protein in 130-170 µL formulation buffer (50 mM sodium acetate/200 mM sucrose + 0.01% Tween-20, pH = 5.4-5.6) were injected intravenously (i.v.) into the tail vein under anesthesia with inhalation of 2-4% isoflurane/O_2_. All mice were anesthetized, bled, euthanized and dissected 7 days p.i. For all mice, blood and organs of interest were collected, weighed and the amount of radioactivity in each sample was measured in a gamma counter (LKB Wallac Gamma Counter, model 1282 Compugamma CS). The brain was dissected into the two hemispheres, using the left cerebral hemisphere for measuring the brain uptake, whilst the right cerebral hemisphere was used for immunohistochemistry and autoradiography. The radioactive uptake was calculated as the percentage of injected dose per gram of tissue (%ID/g), subtracting the uptake in the tail from the total amount of injected activity.

### *Ex vivo* Autoradiography

During animal dissection, the right cerebral hemisphere was flash-frozen in isopentane at -30 °C. A cryostat-microtome was used to cut the frozen right mouse brain hemispheres in 20 µm sections, which were mounted on gelatinized glass slides. Sections were exposed for 3-4 days for the ^125^I groups and 2 weeks for the ^89^Zr groups on a phosphor screen BAS-IP SR 2040 E (General Electric, Eindhoven, the Netherlands). After exposure, the plates were scanned using a Typhoon FLA 7000 imager (General Electric, Eindhoven, the Netherlands).

### Immunofluorescence staining

After the exposure, the same sagittal sections used for autoradiography were fixed in cold acetone (approx. -15 °C), quickly dried under a fan and blocked with 20% normal goat serum for 1 hour at RT. After disposing the goat serum, the tissues were incubated with a Goat anti-human IgG [H+L] Cross-Adsorbed Secondary HRP-Antibody (Invitrogen; 0.4 µg/mL, 1:2000) at RT for 1 hour under dark conditions. After disposing of the antibody solution, the tissue was washed three times with 0.05% Tween-20 in PBS for 5 minutes each, followed by a final wash step with deionized water (dH_2_O) for 5 minutes. Subsequently, the tissue was incubated with a 0.125% freshly filtered Thioflavin S solution at RT for 8 minutes under dark conditions. After disposing the Thioflavin S solution, the tissue was washed for 3 minutes each in the following order; 2x80% EtOH, 1x90% EtOH, 3xdH_2_O. The tissues were mounted with ProLong™ Gold Antifade Mountant (Invitrogen™, P36930). Images of the stained sections were taken with a fluorescence microscope (Zeiss Axio Observer with a Colibri 7 LED light source and an Axiocam 506 monochrome camera) and equally processed using the Zen blue software Version 3.4.

### PET imaging

The PET imaging was performed with dedicated small animal NanoPET/CT and NanoPET/MR scanners (Mediso Ltd., Hungary), equipped with identical PET components. Mice were anesthetized by inhaling 2-4% isoflurane/O_2_ during the whole scanning period (1-h duration per time point). A 5-min CT scan was acquired prior to each PET scan and used for attenuation and scatter correction purposes. Reconstruction was performed using a 3-dimensional reconstruction algorithm (Tera-Tomo; Mediso Ltd.) with four iterations and six subsets, resulting in an isotropic 0.4-mm voxel dimension. Radioactivity uptake was calculated as the percentage of the injected dose per gram of tissue (%ID/g) with the decay-corrected amount of injected radiolabeled compound. Images were analyzed and quantified using the VivoQuant software (Invicro, Boston, USA), and region of interest (ROI) were applied using the VivoQuant-integrated brain atlas tool.

### Statistics

The Grubbs outlier test was used to check and remove outliers. Statistical analysis for **Figure 3** and** Figure 6** and **Table S2-S4** were performed on the organ uptake values of the different groups of mice with the Brown-Forsythe and Welch ANOVA multiple comparison test. A normal Gaussian distribution of the values and no equal variances between groups were assumed. In addition, a Dunnet's T3correction for multiple comparisons was performed. Statistical analysis for **Figure 5** and **Figure 7** were performed on the brain uptake values of the corresponding groups of mice with the Welch's t-test. Significance levels were calculated, and p < 0.05 was considered to be statistically significant. All graphs were generated using GraphPad Prism 9.10 software.

## Supplementary Material

Supplementary methods, figures and tables.

## Figures and Tables

**Table 1:** Labeling results of the radioimmunoconjugates

Type of modification/radiolabeling	Radiochemical yield (%)	Radiochemical purity (%)	Specific activity (MBq µg^-1^)	Injected dose (MBq)
[^89^Zr]Zr-DFO-*N*-suc-mAb	63-67	≥98*	0.19-0.21	5.8 ±0.2
[^89^Zr]Zr-DFO-NCS-mAb	85-92	≥96**		5.8 ±0.2
[^89^Zr]Zr-DFO*-NCS-mAb	83	≥98**		6.7 ±0.1
[^125^I]I-mAb	82-83	≥99*	0.08-0.09	2.6 ±0.1

*Determined by iTLC **Determined by spin filter analysis.

## Abbreviations

AD: Alzheimer's disease
Adu: anti-amyloid-beta aducanumab derivate
BBB: blood-brain barrier
CHO-S: Chinese hamster ovary cells in suspension
CNS: central-nervous-system
CSF: Cerebrospinal fluid
DFO: desferrioxamine
ELISA: enzyme-linked immunosorbent assay
FACS: fluorescence-activated cell sorting
FDA: u.s. food and drug administration
HIV: human immunodeficiency virus
ID: injected dose
immune-PET: immuno-positron emission tomography
iTLC: instant thin-layer chromatography
mAb: monoclonal antibody
mTfR1: murine transferrin receptor 1
p.i.: post injection
RMT: receptor-mediated transcytosis
scFab: single chain fab fragment
scFv: single chain variable fragment
SD: standard deviation
SE-HPLC: size-exclusion high-performance liquid chromatography
suc: succinyl
TG: transgenic
WT: wild type
