# Supplementary material for: Advancing 89Zr-immuno-PET in neuroscience with a bispecific anti-amyloid-beta monoclonal antibody - The choice of chelator is essential
Source: Theranostics. 2022 Oct 9;12(16):7067–79. doi: 10.7150/thno.73509 (PMC9576608; doi:10.7150/thno.73509)
Supplement: Supplementary file 1 — Supplementary methods, figures and tables. [file thnov12p7067s1.pdf]

Advancing ^{89}Zr-immuno-PET in neuroscience with a bispecific anti-amyloid-beta monoclonal antibody - The choice of chelator is essential.

Thomas E. Wuensche[1], Natascha Stergiou[1], Iris Mes[1], Mariska Verlaan[1], Maxime Schreurs[1], Esther J. M. Kooijman[1], Bart Janssen[1], Albert D. Windhorst[1,2], Allan Jensen[3], Ayodeji A. Asuni[3], Benny Bang-Andersen[3], Wissam Beaino[1,2], Guus A. M. S. van Dongen[1,2], Danielle J. Vugts[1,2]

[1] *Amsterdam UMC location Vrije Universiteit Amsterdam, dept Radiology & Nuclear Medicine, De Boelelaan 1117, Amsterdam, The Netherlands*

[2] *Amsterdam Neuroscience, Brain imaging, Amsterdam, The Netherlands*

[3] *Lundbeck A/S, Valby, Denmark*

First Author: t.wunsche@amsterdamumc.nl

Corresponding Author: d.vugts@amsterdamumc.nl

SUPPLEMENTARY DATA

Antibody constructs

All antibodies were produced by H. Lundbeck A/S in Valby, Denmark. The monospecific antibody aducanumab (Adu) targets insoluble amyloid-beta plaques and soluble aggregates, e.g., protofibrils/oligomers, but not amyloid-beta monomers. The bispecific antibody Adu-8D3 was also designed with the scFab8D3 moiety, fused on the heavy chain C terminus, to target the murine TfR1 and allow shuttling across the BBB. To assess non-specific uptake, B12-8D3 was produced and used as a control antibody, targeting gp120 of HIV1.

Antibody design

All antibodies are built on the human IgG1 framework and hold Fc-null mutations K322A, L234A, and L235A, as described by Lin *et al*. and Hezareh *et al*. [1,2] to abolish binding to FcgR. Bispecific mAbs with monospecific murine TfR1 binding (scFab8D3) were generated using the Knobs-in-Hole technology. On the Knob heavy chain (HC), a 6xG4S linker followed by the 8D3 scFab extension was engineered at the C-terminal. The Hole HC has an average Fc length.

Cell culture

Synthetic genes of the heavy chain (HC) and light chain (LC) optimized with human codon composition were sub-cloned into the pTT5 vector for transient expression. Transfection of HC and LC expression vectors was performed in HEK293 6E cells using PEIpro (Polyplus) as a transfection reagent. The HEK293 6E expression system, including the pTT5 vector, is licensed by the National Research Council of Canada (NRCC). Transfected cells were cultured until the viability had dropped to around 50 %, and culture media was harvested by centrifugation and sterile-filtration and kept cold (4 °C) until purification.

Purification

Antibodies in the cell culture harvest were purified by capturing a HiTrap protein G (Cytiva) followed by washing with PBS and elution with 0.1 M Glycine pH 2.7. After dialysis against 20 mM Tris pH 7.5, the sample was passed through a Q-sepharose column (Cytiva) and equilibrated with the same buffer. The flow-through is concentrated to <5 ml and separated on a HiLoad® 16/600 Superdex® (Cytiva) in PBS. Fractions were analyzed by SDS-PAGE, SEC, and LC-MS. Selection for pooling was made to minimize aggregates, incorrectly paired molecules, and free LC. After purification, a certain amount of Hole dimer (double Hole HC with average Fc length) was still present in the used Adu-8D3 and B12-8D3 batches (**Figure S7 and S8**; t_R= 16.5/17.6; ~20%)

Biodistribution

Table S1: Summary of all *in vivo* study groups; including number, sex, the weight of the mice, and corresponding *ex vivo* brain uptake day 7 p.i. by biodistribution. Results are expressed as mean (%ID/g) ±standard deviation (sd).

Protein	Mice	n Mice	Age in months	Average weight in gram	Brain uptake %ID/g by BD	Manuscript figure:	Complete biodistribution:
[^{89}Zr]Zr-DFO-*N*-suc-Adu-8D3	TG male	4	13	34.15 ±4.9	**0.61 ±0.11**	3, 5	Table S2, Figure S1
[^{89}Zr]Zr-DFO-*N*-suc-B12-8D3	TG male	4	13	46.63 ±5.9	**0.32 ±0.06**	3, 5	Table S3, Figure S2
[^{89}Zr]Zr-DFO-NCS-Adu-8D3	TG female	4	11	29.33 ±3.0	**0.60 ±0.18**	3, 5	Table S2, Figure S1
[^{89}Zr]Zr-DFO-NCS-B12-8D3	TG female	3	11	35.66 ±0.5	**0.44 ±0.06**	3, 5	Table S3, Figure S2
[^{89}Zr]Zr-DFO*-NCS-Adu-8D3	TG female	4	11	26.03 ±2.5	**2.19 ±0.12**	3, 5	Table S2, Figure S1
	TG male	4	13	35.66 ±2.9	**1.73 ±0.14**	6,7	Table S4, Figure S3
	WT male	5	13	40.34 ±4.7	**0.78 ±0.10**	6,7	Table S4, Figure S3
[^{89}Zr]Zr-DFO*-NCS-B12-8D3	TG female	4	11	27.85 ±2.4	**0.86 ±0.02**	3, 5	Table S3, Figure S2
[^{125}I]I-Adu-8D3	TG female	4	11	29.45 ±5.8	**2.21 ±0.15**	3, 5	Table S2, Figure S1
	TG male	4	13	44.28 ±9.4	**1.74 ±0.17**	6,7	Table S4, Figure S3
	WT male	5	13	39.62 ±6.8	**0.05 ±0.02**	6,7	Table S4, Figure S3
[^{125}I]I-B12-8D3	TG female	4	11	29.73 ±8.8	**0.04 ±0.01**	3, 5	Table S3, Figure S2
[^{125}I]I-Adu	TG male	5	13	42.63 ±4.8	**0.25 ±0.03**	-	Table S5, Figure S4

Table S2: Biodistribution of [^{89}Zr]Zr-DFO-*N*-suc-Adu-8D3, [^{89}Zr]Zr-DFO-NCS-Adu-8D3, [^{89}Zr]Zr-DFO*-NCS-Adu-8D3, and [^{125}I]I-Adu-8D3 in APP/PS1 TG mice, 7 days after injection of 30 µg radiolabeled protein. Results are expressed as mean (%ID/g) ±sd (n=4-5 mice per group). Significant differences between the groups are marked with asterisks (*p < 0.05; **p < 0.01; ***p < 0.001; ****p < 0.0001) or marked as non-significant (ns).

7 days p.i.	I) [^{89}Zr]Zr-DFO-*N*-suc-Adu-8D3<br>male APP/PS1 TG mice, 13 months old	II) [^{89}Zr]Zr-DFO-NCS-Adu-8D3<br>female APP/PS1 TG mice 11 months old	III) [^{89}Zr]Zr-DFO*-NCS-Adu-8D3<br>female APP/PS1 TG mice 11 months old	IV) [^{125}I]I-Adu-8D3<br>female APP/PS1 TG mice 11 months old	Summary of Dunnett's T3 multiple comparisons test
blood	0.36 ±0.1	0.40 ±0.05	0.55 ±0.07	0.42 ±0.09	-
urine	0.36 ±0.1	0.37 ±0.13	1.07 ±0.39	0.53 ±0.06	-
skin	0.62 ±0.1	0.58 ±0.18	0.42 ±0.09	0.22 ±0.05	*I/IV
bladder	1.29 ±0.2	0.81 ±0.09	0.95 ±0.21	0.38 ±0.05	*I/IV; **II/IV
sternum	4.67 ±0.9	2.82 ±0.57	1.20 ±0.23	0.11 ±0.03	*I/II, I/IV, II/IV, III/IV
heart	0.79 ±0.1	0.63 ±0.06	0.43 ±0.04	0.20 ±0.06	*I/IV, III/IV, II/III; **II/III
lung	1.06 ±0.2	0.91 ±0.35	0.60 ±0.08	0.27 ±0.08	*I/IV, III/IV
liver	10.53 ±3.2	16.34 ±1.81	10.69 ±1.26	0.15 ±0.03	*I/IV, II/III; **II/IV, III/IV
pancreas	0.67 ±0.0	0.33 ±0.10	0.16 ±0.02	0.07 ±0.02	*III/IV; ****I/III, I/IV
spleen	75.45 ±15.6	231.85 ±54.09	45.56 ±5.80	1.39 ±0.26	*I/IV, II/IV, II/III; **III/IV
kidney l	5.60 ±0.6	6.95 ±0.65	4.26 ±0.41	0.24 ±0.04	*II/III; **I/II, I/III, I/IV
muscle	0.20 ±0.0	0.12 ±0.01	0.11 ±0.01	0.05 ±0.01	**I/II, I/III, II/IV, III/IV; ***I/IV
thigh bone	13.31 ±1.9	7.56 ±0.10	4.00 ±0.33	0.18 ±0.04	*I/III; **I/IV, III/IV; ***II/IV; ****II/IV
colon	0.49 ±0.0	0.52 ±0.10	0.66 ±0.05	0.09 ±0.01	*I/III, II/IV; **III/IV; ***I/IV
colon content	0.24 ±0.1	0.32 ±0.06	0.45 ±0.05	0.05 ±0.01	*I/III, I/IV, II/IV; **III/IV
ileum	0.90 ±0.1	0.77 ±0.08	0.98 ±0.09	0.12 ±0.03	**I/IV, II/IV, III/IV
ileum content	0.43 ±0.2	0.67 ±0.28	0.31 ±0.07	0.04 ±0.01	*III/IV
stomach	0.56 ±0.1	0.41 ±0.02	0.36 ±0.02	0.14 ±0.03	**I/IV; ***II/IV, III/IV
stomach content	0.06 ±0.0	0.03 ±0.02	0.05 ±0.02	0.14 ±0.06	-
tail	1.68 ±0.4	2.72 ±1.32	1.36 ±0.31	0.25 ±0.04	*I/IV, III/IV
spine	11.59 ±1.4	3.25 ±0.26	0.92 ±0.08	0.17 ±0.01	**all
Thyroid	-	0.75 ±0.25	0.63 ±0.10	103.72 ±41.08	-

Table S3: Biodistribution of [^{89}Zr]Zr-DFO-*N*-suc-B12-8D3, [^{89}Zr]Zr-DFO-NCS-B12-8D3, [^{89}Zr]Zr-DFO*-NCS-B12-8D3 and [^{125}I]I-B12-8D3 in APP/PS1 TG mice, 7 days after injection of 30 µg radiolabeled protein. Results are expressed as mean (%ID/g) ±sd (n=3-5 mice per group). Significant differences between the groups are marked with asterisks (*p < 0.05; **p < 0.01; ***p < 0.001; ****p < 0.0001) or marked as non-significant (ns).

7 days p.i.	I) [^{89}Zr]Zr-DFO-*N*-suc-B12-8D3<br>male APP/PS1 TG mice, 13 months old	II) [^{89}Zr]Zr-DFO-NCS-B12-8D3<br>female APP/PS1 TG mice 11 months old	III) [^{89}Zr]Zr-DFO*-NCS-B12-8D3<br>female APP/PS1 TG mice 11 months old	IV) [^{125}I]I-B12-8D3<br>female APP/PS1 TG mice 11 months old	Summary of Dunnett's T3 multiple comparisons test
blood	0.49 ±0.2	0.74 ±0.15	1.08 ±0.10	0.69±0.05	*I/III, III/IV
urine	0.31 ±0.1	3.05 ±2.97	0.74 ±0.11	0.29±0.14	*I/III
skin	1.44 ±0.7	0.48 ±0.26	0.35 ±0.05	0.23±0.06	*I/IV
bladder	3.10 ±3.8	1.30 ±0.10	0.87 ±0.11	0.23±0.05	**II/IV, III/IV
sternum	3.54 ±2.1	3.63 ±0.03	1.42 ±0.14	0.10±0.01	**III/IV; ***II/III; ****II/IV
heart	0.85 ±0.1	0.96 ±0.07	0.53 ±0.03	0.21±0.02	*I/III, II/IV; **I/IV; ***III/IV
lung	0.89 ±0.1	1.24 ±0.22	0.79 ±0.06	0.29±0.03	*I/IV; ***III/IV
liver	9.83 ±0.9	21.73 ±1.45	7.20 ±1.22	0.15±0.03	*I/II, III/IV; **I/IV, II/III, II/IV
pancreas	0.51 ±0.1	0.82 ±0.16	0.20 ±0.02	0.08±0.01	*I/III, I/IV; **III/IV
spleen	50.44 ±16.7	313.80 ±66.62	27.36 ±5.00	0.38±0.09	*III/IV
kidney l	2.78 ±0.5	4.54 ±0.18	2.35 ±0.27	0.32±0.06	*I/II, I/IV; **II/IV, III/IV
muscle	0.34 ±0.1	1.92 ±2.08	0.11 ±0.01	0.05±0.01	*I/IV, III/IV
thigh bone	8.56 ±2.6	6.52 ±3.80	3.72 ±0.18	0.10±0.01	*I/IV; ***III/IV
colon	0.33 ±0.2	0.65 ±0.03	0.61 ±0.11	0.09±0.02	*III/IV; **II/IV
colon content	0.39 ±0.1	0.93 ±0.17	0.59 ±0.12	0.04±0.01	*I/IV, III/IV
ileum	0.47 ±0.1	1.22 ±0.06	0.90 ±0.08	0.09±0.01	*I/IV, II/III; **I/III, II/IV, III/IV; ***I/II
ileum content	0.18 ±0.1	1.06 ±0.37	0.34 ±0.10	0.06±0.01	-
stomach	0.38 ±0.1	1.12 ±0.61	0.47 ±0.05	0.18±0.03	**III/IV
stomach content	0.04 ±0.0	0.06 ±0.02	0.11 ±0.01	0.45±0.08	*I/III
tail	1.73 ±0.8	1.42 ±0.29	0.80 ±0.18	0.20±0.04	*III/IV
spine	7.86 ±2.1	4.54 ±0.16	0.93 ±0.05	0.08±0.01	*I/III, I/IV; **II/III, II/IV; ***III/IV
Thyroid	-	1.15 ±0.29	0.64 ±0.13	29.14±12.34	-

Table S4: Biodistribution of [^{89}Zr]Zr-DFO*-NCS-Adu-8D3 and [^{125}I]I-Adu-8D3 in APP/PS1 TG or WT mice, 7 days after injection of 30 µg radiolabeled protein. Results are expressed as mean (%ID/g) ±sd (n=4-5 mice per group). Significant differences between the groups are marked with asterisks (*p < 0.05; **p < 0.01; ***p < 0.001; ****p < 0.0001) or marked as non-significant (ns).

7 days p.i.	<u>I)</u> [^{89}Zr]Zr-DFO*-NCS-Adu-8D3<br>male APP/PS1 TG mice, 11 months old	<u>II)</u> [^{89}Zr]Zr-DFO*-NCS-Adu-8D3<br>male WT mice, 11 months old	<u>III)</u> [^{125}I]I-Adu-8D3<br>male APP/PS1 TG mice, 11 months old	<u>IV)</u> [^{125}I]I-Adu-8D3<br>male WT mice, 11 months old	Summary of Dunnett's T3 multiple comparisons test
blood	0.41 ±0.1	0.55 ±0.1	0.43 ±0.01	0.46±0.1	-
urine	0.66 ±0.1	0.57 ±0.1	0.37 ±0.07	0.39±0.1	*I/IV; **I/III
skin	0.66 ±0.1	0.40 ±0.1	0.27 ±0.07	0.30±0.1	*I/III, I/IV
bladder	0.67 ±0.0	0.74 ±0.1	0.27 ±0.05	0.28±0.1	***I/III, II/III, II/IV; ****I/IV
sternum	1.06 ±0.1	1.07 ±0.2	0.11 ±0.03	0.11±0.0	**I/IV, II/III, II/IV; ***I/III
heart	0.33 ±0.1	0.36 ±0.0	0.11 ±0.04	0.14±0.1	*I/III, I/IV; II/IV, ***II/III
lung	0.45 ±0.1	0.61 ±0.1	0.20 ±0.06	0.23±0.1	*I/II, I/IV; **I/III; ***II/III, II/IV
liver	4.73 ±0.7	5.95 ±0.5	0.13 ±0.02	0.13±0.0	**I/III, I/IV; ***II/III, II/IV
pancreas	0.13 ±0.0	0.19 ±0.1	0.04 ±0.01	0.06±0.0	*I/IV; **I/III
spleen	55.02 ±11.5	34.79 ±	1.22 ±0.14	1.21±0.3	*I/III, I/IV; **II/III, II/IV
kidney l	1.80 ±0.2	2.34 ±0.3	0.34 ±0.06	0.42±0.2	**II/III; ***I/III, I/IV, II/IV
muscle	0.10 ±0.0	0.10 ±0.0	0.06 ±0.00	0.05±0.0	*I/III, I/IV; **II/III, II/IV
thigh bone	2.94 ±0.6	3.48 ±0.4	0.20 ±0.06	0.18±0.0	*I/III, I/IV; ***II/III, II/IV
colon	0.51 ±0.1	0.50 ±0.1	0.08 ±0.02	0.08±0.0	*I/III, I/IV, II/III, II/IV
colon content	0.44 ±0.1	0.62 ±0.0	0.04 ±0.00	0.04±0.0	*I/III, I/IV; ****II/III, II/IV
ileum	0.81 ±0.2	1.07 ±0.2	0.10 ±0.02	0.16±0.1	*I/III, I/IV; **II/III, II/IV
ileum content	0.32 ±0.1	0.51 ±0.1	0.04 ±0.01	0.04±0.0	*I/III, I/IV; **II/III, II/IV
stomach	0.33 ±0.1	0.36 ±0.1	0.12 ±0.01	0.13±0.0	*I/II, I/IV; **II/III, II/IV
stomach content	0.09 ±0.0	0.20 ±0.0	0.16 ±0.02	0.15±	*I/III; **I/II
tail	2.00 ±1.0	1.68 ±0.8	0.45 ±0.19	0.29±	-
spine	1.26 ±0.1	1.50 ±0.2	0.16 ±0.04	0.11±	**I/IV; ***I/III, II/III, II/IV
Thyroid and Trachea	0.48 ±0.0	0.65 ±0.1	-	33.33±	*I/II; **I/IV, II/IV

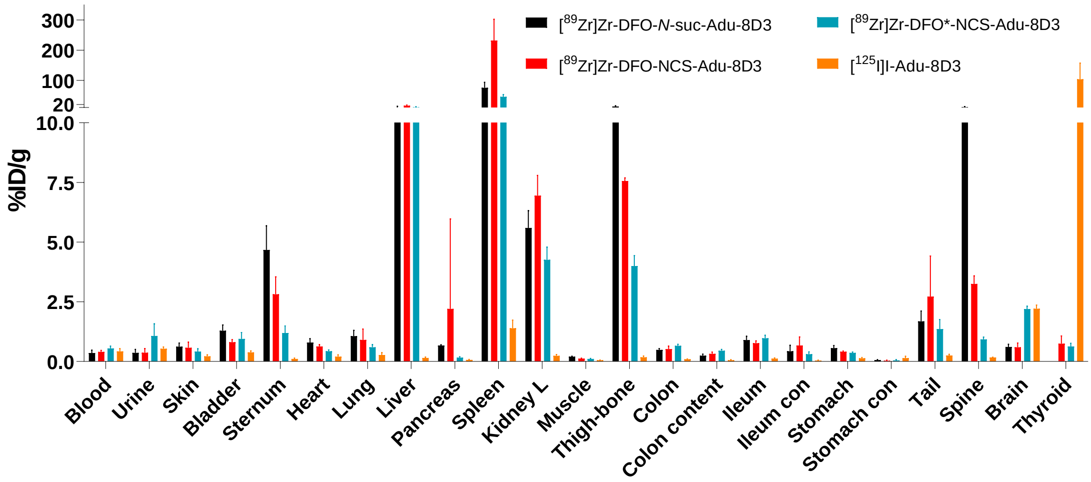

Figure S1:Corresponding biodistribution to Table S2 in APP/PS1 TG mice, 7 days after injection of 30 µg radiolabeled protein. Results are expressed as mean (%ID/g) ±sd (n=4-5 mice per group)

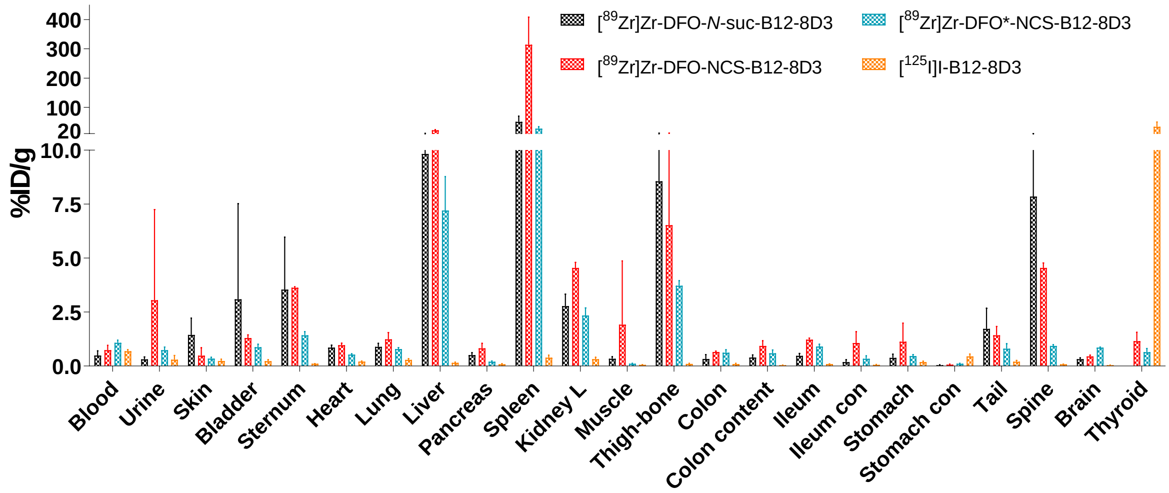

Figure S2: Corresponding biodistribution to Table S3 in APP/PS1 TG mice, 7 days after injection of 30 µg radiolabeled protein. Results are expressed as mean (%ID/g) ±sd (n=3-5 mice per group)

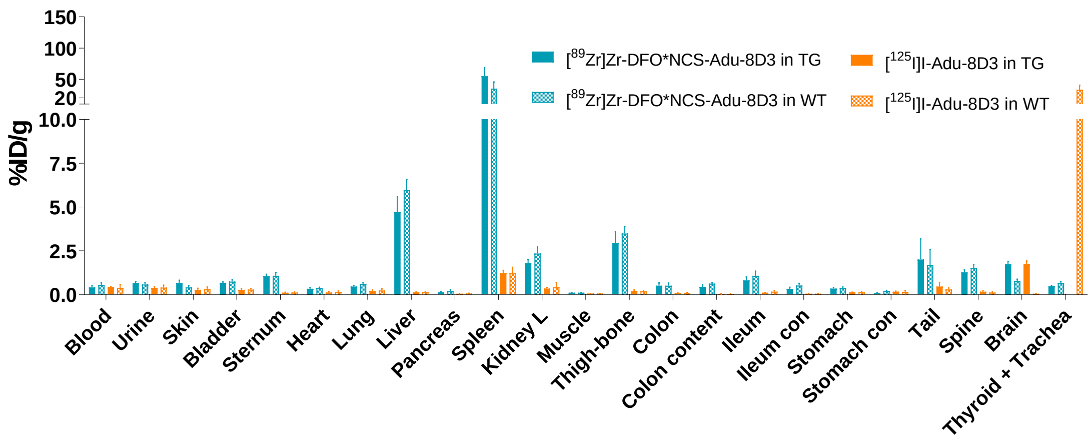

Figure S3: Corresponding biodistribution to Table S4 in APP/PS1 TG or WT mice, 7 days after injection of 30 µg radiolabeled protein. Results are expressed as mean (%ID/g) ±sd (n=4-5 mice per group).

Table S5: Biodistribution of [^{125}I]I-Adu in APP/PS1 TG mice, 7 days after injection of 30 µg radiolabeled protein. Results are expressed as mean (%ID/g) ±sd (n=4 mice).

**7 days p.i.**	**[^{125}I]I-Adu male APP/PS1 TG mice, 11 months old**		
blood	3.24	±	0.4
urine	0.87	±	0.4
skin	1.23	±	0.1
bladder	2.66	±	0.5
sternum	0.78	±	0.1
heart	1.31	±	0.1
lung	2.54	±	0.4
liver	0.73	±	0.1
pancreas	0.57	±	0.1
spleen	1.44	±	0.2
kidney l	1.90	±	0.2
muscle	0.42	±	0.0
thigh bone	0.82	±	0.1
colon	0.95	±	0.1
colon content	0.08	±	0.0
ileum	1.51	±	0.6
ileum content	0.28	±	0.1
stomach	1.04	±	0.1
stomach content	0.28	±	0.1
tail	2.32	±	0.7
spine	0.84	±	0.1
brain	0.25	±	0.0
Thyroid	54.47	±	14.1

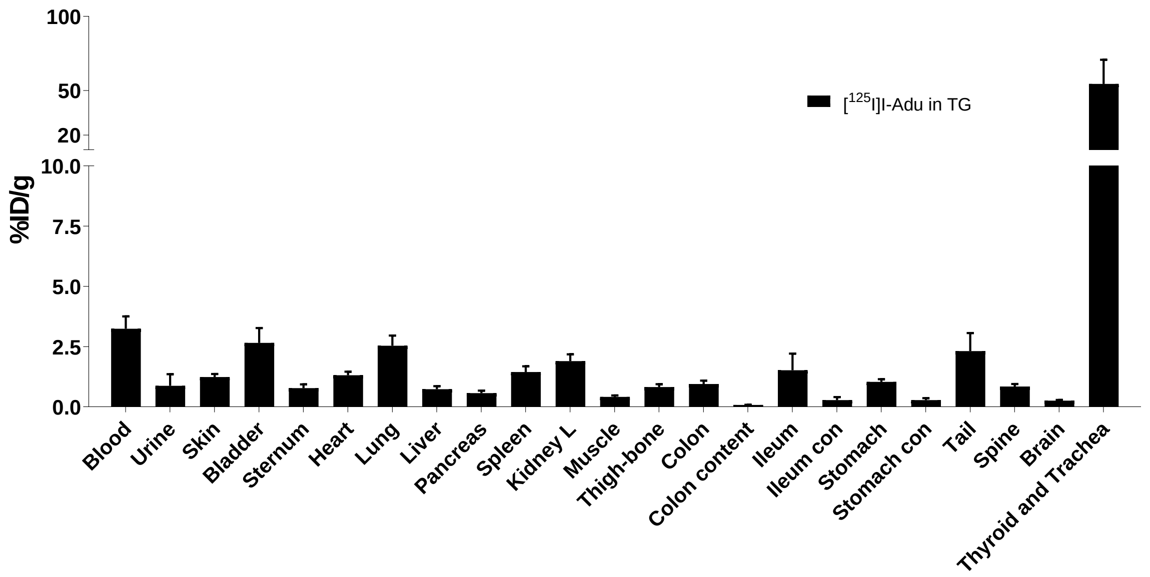

Figure S4: Corresponding biodistribution to Table S5 in APP/PS1 TG mice, 7 days after injection of 22.5 µg radiolabeled protein. Results are expressed as mean (%ID/g) ±sd (n=4 mice).

Immunofluorescence staining

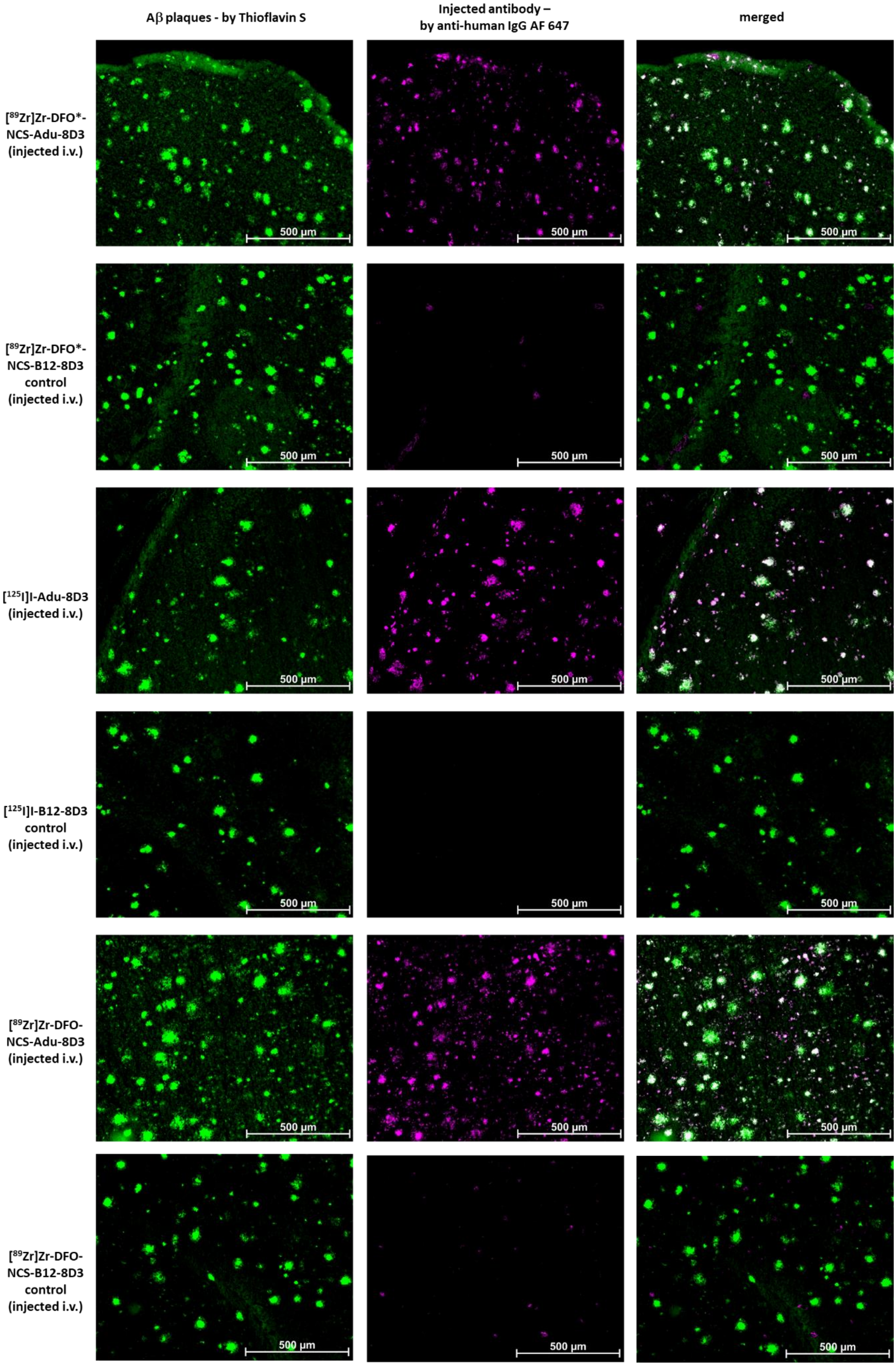

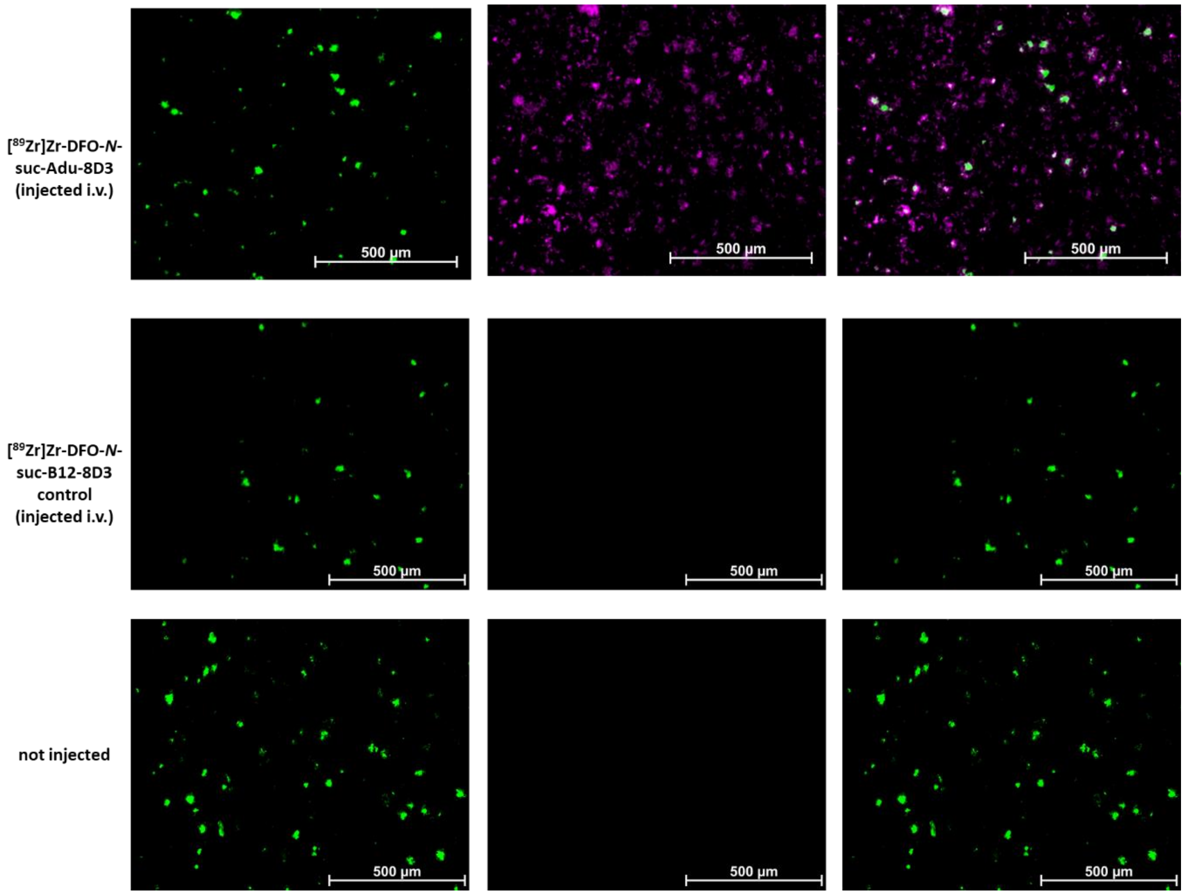

Figure S5: Immunofluorescence analysis of brain sections of APP/PS1 mice. 11-13 month old APP/PS1 TG mice were injected with a fixed dose of 30 µg of radiolabeled protein. The same 20 µm cryo-sections used for autoradiography (Figure 3) were stained with 0.125% Thioflavin S (yellow) and AF647-goat anti-human IgG (1:1000, purple) to detect injected antibody. Shown are the images of each separate and the merged channels, overlay of the two signals appears in white.

Quality controls – Size-exclusion high-performance liquid chromatography

Protein concentration was determined by size-exclusion high-performance liquid chromatography (SE-HPLC). In short, a Jasco or Shimadzu HPLC system was equipped with a Superdex® 200 Increase 10/300 GL (30 cm × 10 mm, 8.6 µm) size exclusion column (GE Healthcare Life Sciences) and a guard column using 0.05 M phosphate buffer/0.15 M NaCl/0.01 M NaN_3 (pH = 6.7) as mobile phase with a run time of 40 min at 0.75 mL/min. Antibody concentration was assessed using the areas under the curve on the UV channel at 280 nm. The concentration was determined against a calibration curve of the unlabeled compound. All three used unmodified antibodies are shown in **Figure S6-S8** and [^{89}Zr]Zr-DFO*-NCS-Adu-8D3 as an example in **Figure S9**.

SE-HPLC chromatograms of mAbs

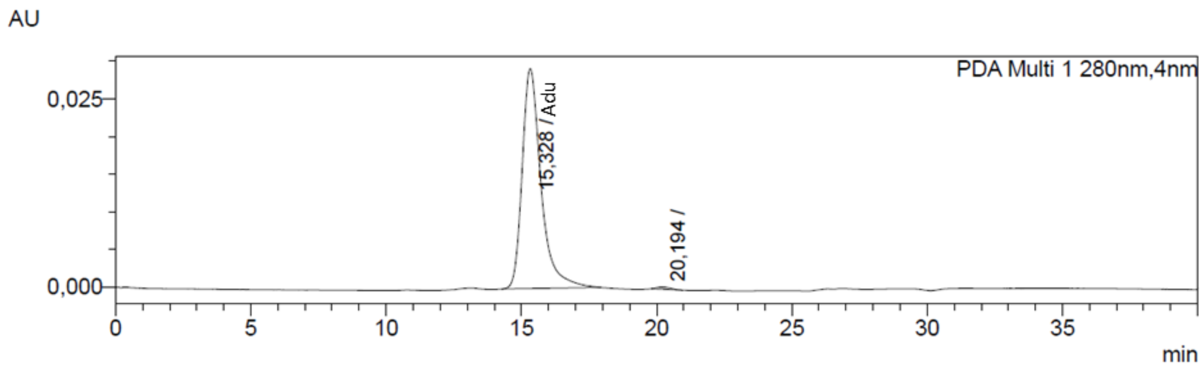

Figure S6: SE-HPLC chromatogram (UV absorption at 280 nm) of Adu.

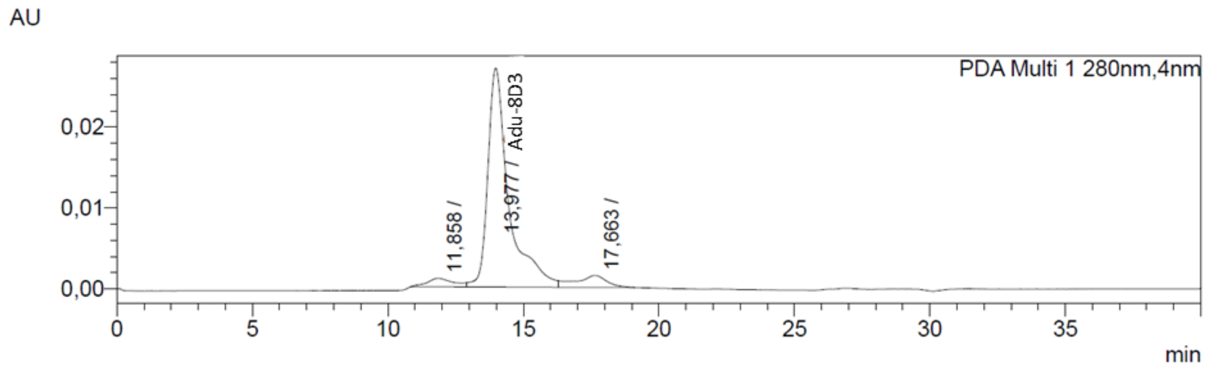

Figure S7: SE-HPLC chromatogram (UV absorption at 280 nm) of Adu-8D3.

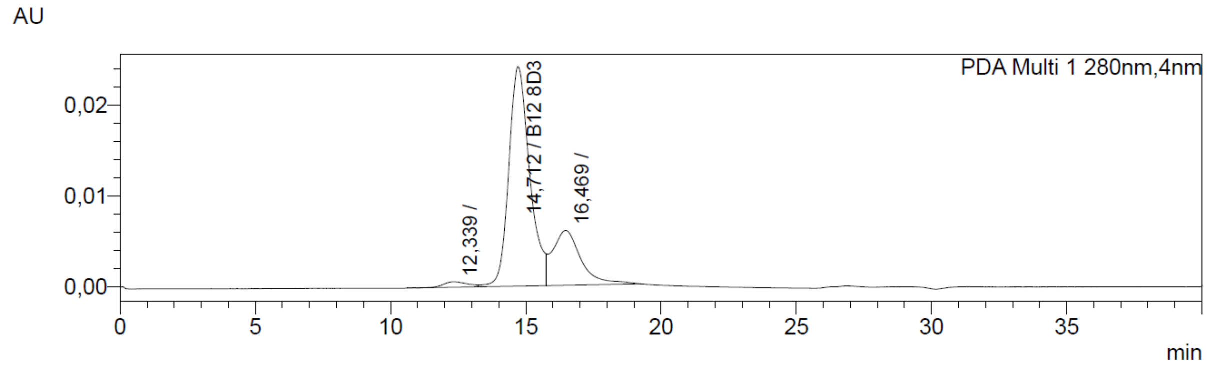

Figure S8: SE-HPLC chromatogram (UV absorption at 280 nm) of B12-8D3.

SE-HPLC chromatograms of radiolabeled [^{89}Zr]Zr-DFO-NCS-Adu-8D3*

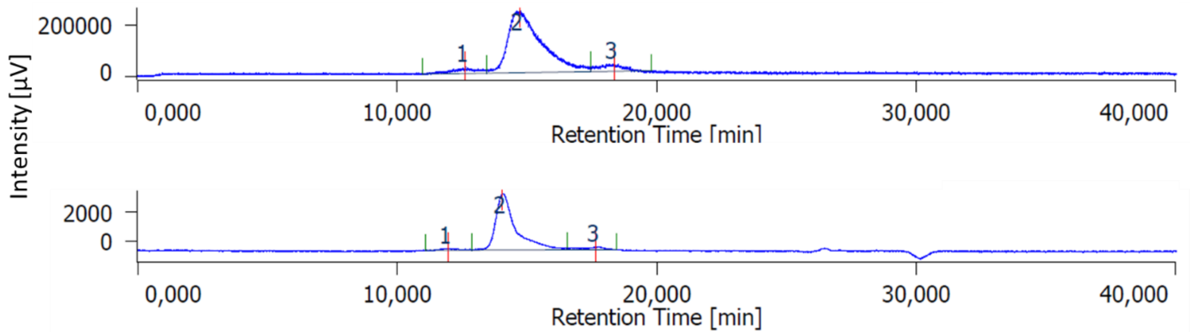

Figure S9: SE-HPLC chromatogram of **[^{89}Zr]Zr-DFO*-NCS-Adu-8D3** after formulation (unlabeled Adu-8D3 and buffer added). Top panel: radiodetector, bottom panel: UV absorption at 280 nm.

Determination of the chelator-to-mAb ratio

To ensure high specific activity, we adapted our standard conjugation procedures by using higher equivalents of DFO-NCS, DFO*-NCS, or TFP-*N*-suc-DFO-Fe-ester during mAb conjugation, with longer reaction times and smaller reaction volumes to achieve a chelator-to-mAb ratio of around 2.5:1.

For the TFP-*N*-suc-DFO-Fe-ester modification of Adu-8D3, a sample was taken before the Fe-removal step and a SE-HPLC analysis was performed to determine the chelator-to-mAb ratio [3]. The ratio of the two signals from Fe-*N*-suc-DFO-Adu-8D3 and unreacted TFP-*N*-suc-DFO-Fe-ester at 430 nm was used to determine a chelator-to-mAb ratio of 2.85.

An SE-MS approach to analyse the chelator-to-mAb ratio, as reported by Sijbrandi *et al.* [4], for the analysis of antibody-drug conjugates did not provide reliable results for the DFO*-NCS and DFO-NCS modified mAb samples due to the thiourea bond in those conjugates as previously reported [5]. Furthermore, to evaluate the chelator-to-mAb ratio, we also performed a previously reported method by Chomet *et al.* [5] in which the chelator-mAb conjugate was <u>not</u> purified from unreacted chelator and radiolabeled with ^{89}Zr (1 MBq). This approach neither led to plausible results, probably because of the considerably higher equivalents (10 eq.) of DFO*-NCS or DFO-NCS used in our labelings. Finally, we considered titration methods as used by Vugts *et al*. [6]. However, this method could not be performed due to the large amounts of antibody needed, which makes this method more suitable for commercially available antibodies. Because of this, we have to rely on calculation for an estimated chelator-to-mAb ratio: with the molar activity of the used ^{89}Zr (20-30 MBq/nmol) and the achieved yields for both NCS modifications with 150-250 MBq ^{89}Zr and 500 µg (2.5 nmol) protein, it can be assumed that a chelator-to-mAb ratio of between 2:1 to 5:1 was reached.

Modification procedures and achieved radiolabelling yields for B12-8D3 and Adu-8D3 were similar, assuming a similar chelator-to-mAb ratio for the respective modification.

FACS analysis - gating

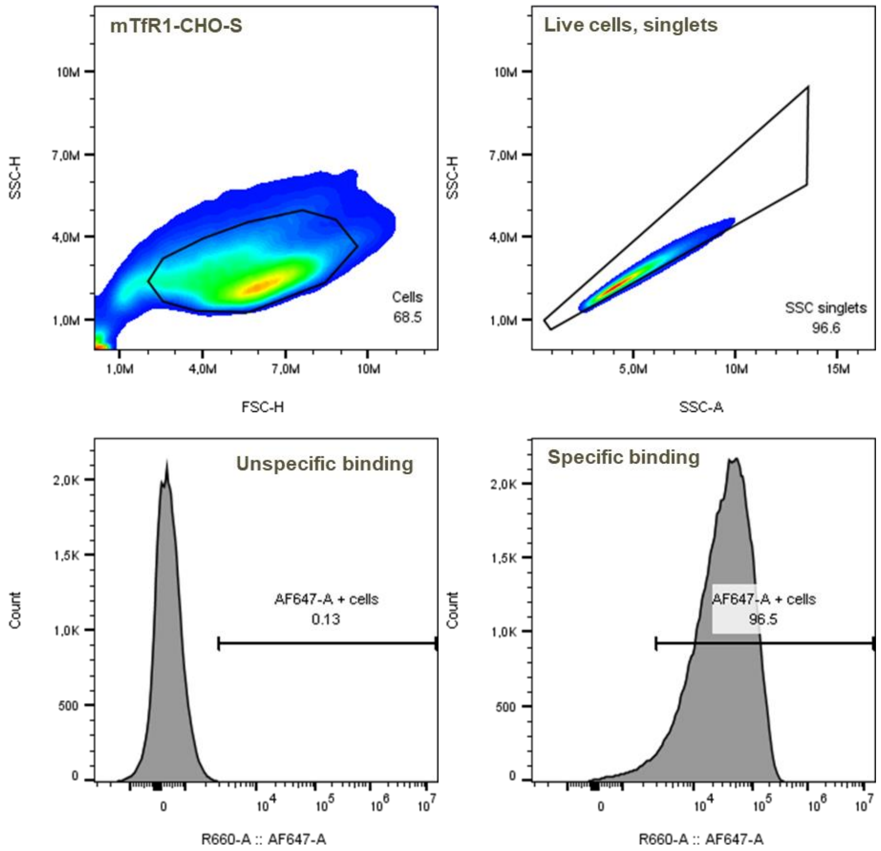

Figure S10: Example figure for the used FACS gating strategy. Cells were gated for removing debris (top left), for live and single cells (top right), and the % of binding (% of positive cells for the antibody tested that is labeled with AF647). The percentage of binding was determined for each mAb and modified mAb in comparison to the unspecific binding of the secondary antibody conjugated with AF647 (bottom left and right) via FlowJo 10 software.

Whole body PET images – day 7 p.i.

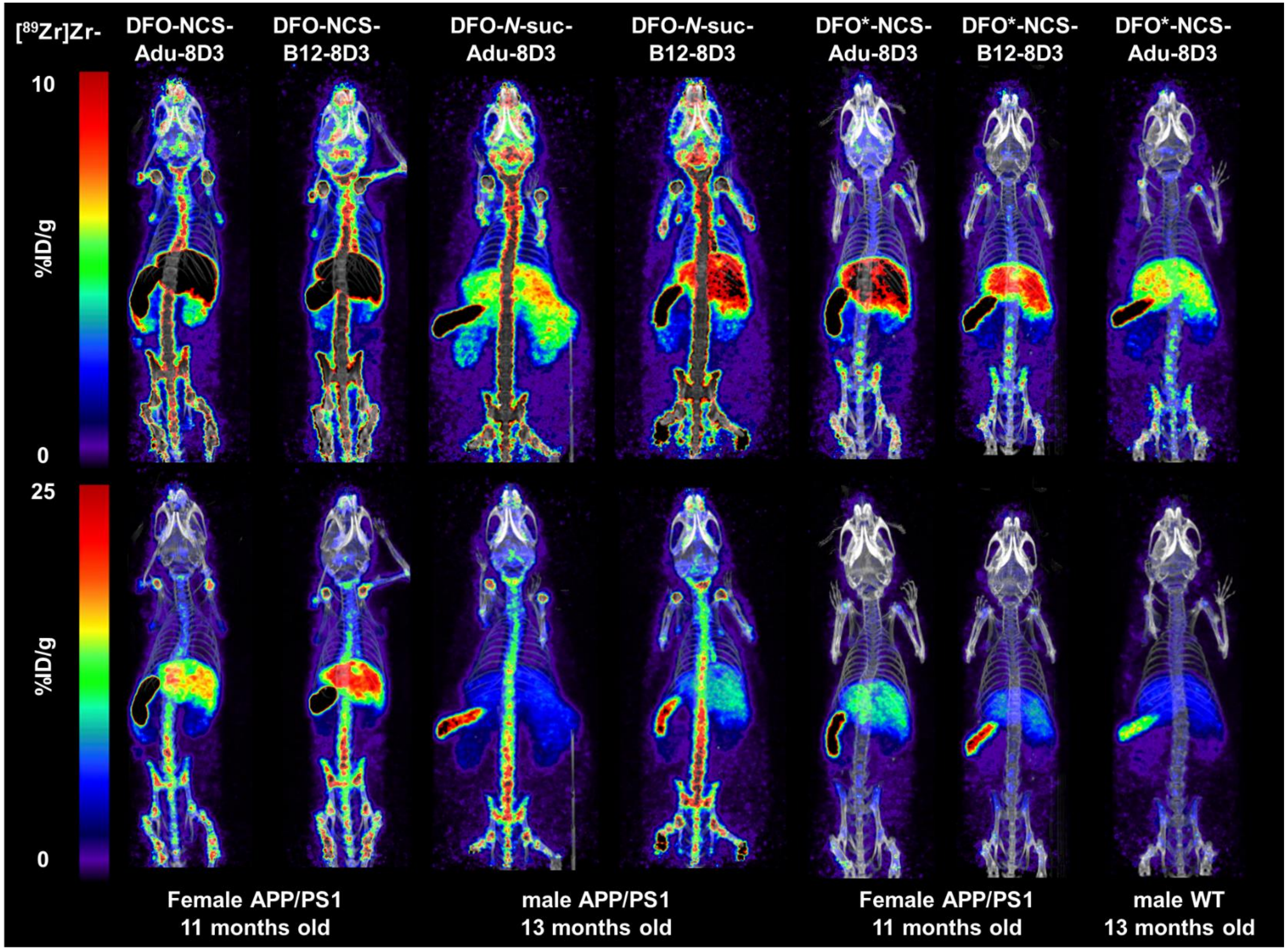

Figure S11: Representative whole body PET/CT MIP images for all in vivo study groups day 7 p.i., injected with 30 µg of the radioimmunoconjugate.

References

1. Lin JM, Spidel JL, Maddage CJ, Rybinski KA, Kennedy RP, Krauthauser CLM, et al. The antitumor activity of the human FOLR1-specific monoclonal antibody, farletuzumab, in an ovarian cancer mouse model is mediated by antibody-dependent cellular cytotoxicity. Cancer Biol Ther. 2013; 14: 1032–8.

2. Hezareh M, Hessell AJ, Jensen RC, van de Winkel JGJ, Parren PWHI. Effector Function Activities of a Panel of Mutants of a Broadly Neutralizing Antibody against Human Immunodeficiency Virus Type 1. J Virol. 2001; 75: 12161–8.

3. Verel I, Visser GWM, Boellaard R, Walsum MS, Snow GB, Dongen GAMS Van, et al. 89Zr Immuno-PET: Comprehensive Procedures for the Production of 89Zr-Labeled Monoclonal Antibodies. J Nucl Med. 2003; 1271–81.

4. Sijbrandi NJ, Merkul E, Muns JA, Waalboer DCJ, Adamzek K, Bolijn M, et al. A Novel Platinum(II)-Based Bifunctional ADC Linker Benchmarked Using Zr-Desferal and Auristatin F Conjugated Trastuzumab. Cancer Res. 2017; 77: 257–67.

5. Chomet M, Schreurs M, Bolijn MJ, Verlaan M, Beaino W, Brown K, et al. Head-to-head comparison of DFO* and DFO chelators: selection of the best candidate for clinical 89Zr-immuno-PET. Eur J Nucl Med Mol Imaging. 2021; 48: 694–707.

6. Vugts DJ, Klaver C, Sewing C, Poot AJ, Adamzek K, Huegli S, et al. Comparison of the octadentate bifunctional chelator DFO*-pPhe-NCS and the clinically used hexadentate bifunctional chelator DFO-pPhe-NCS for 89Zr-immuno-PET. Eur J Nucl Med Mol Imaging. 2017; 44: 286–95.
